# Soybean Stem Canker Caused by *Diaporthe caulivora*; Pathogen Diversity, Colonization Process, and Plant Defense Activation

**DOI:** 10.3389/fpls.2019.01733

**Published:** 2020-01-23

**Authors:** Eilyn Mena, Silvina Stewart, Marcos Montesano, Inés Ponce de León

**Affiliations:** ^1^ Departamento de Biología Molecular, Instituto de Investigaciones Biológicas Clemente Estable, Montevideo, Uruguay; ^2^ Sección Protección Vegetal, Instituto Nacional de Investigación Agropecuaria, La Estanzuela, Uruguay; ^3^ Laboratorio de Fisiología Vegetal, Centro de Investigaciones Nucleares, Facultad de Ciencias, Universidad de la República, Montevideo, Uruguay

**Keywords:** soybean stem canker, *Diaporthe caulivora*, internal transcribed spacer (ITS) ribosomal RNA (rDNA), translation elongation factor 1-alpha gene (TEF1α), disease symptoms, pathogen colonization, cell wall, defense genes

## Abstract

Soybean is an important crop in South America, and its production is limited by fungal diseases caused by species from the genus *Diaporthe*, including seed decay, pod and stem blight, and soybean stem canker (SSC). In this study, we focused on *Diaporthe* species isolated from soybean plants with SSC lesions in different parts of Uruguay. *Diaporthe* diversity was determined by sequencing the internal transcribed spacer (ITS) regions of ribosomal RNA and a partial region of the translation elongation factor 1-alpha gene (TEF1α). Phylogenetic analysis showed that the isolates belong to five defined groups of *Diaporthe* species, *Diaporthe caulivora* and *Diaporthe longicolla* being the most predominant species present in stem canker lesions. Due to the importance of *D. caulivora* as the causal agent of SSC in the region and other parts of the world, we further characterized the interaction of this pathogen with soybean. Based on genetic diversity of *D. caulivora* isolates evaluated with inter-sequence single repetition (ISSR), three different isolates were selected for pathogenicity assays. Differences in virulence were observed among the selected *D. caulivora* isolates on susceptible soybean plants. Further inspection of the infection and colonization process showed that *D. caulivora* hyphae are associated with trichomes in petioles, leaves, and stems, acting probably as physical adhesion sites of the hyphae. *D. caulivora* colonized the stem rapidly reaching the phloem and the xylem at 72 h post-inoculation (hpi), and after 96 hpi, the stem was heavily colonized. Infected soybean plants induce reinforcement of the cell walls, evidenced by incorporation of phenolic compounds. In addition, several defense genes were induced in *D. caulivora*–inoculated stems, including those encoding a pathogenesis-related protein-1 (PR-1), a PR-10, a β-1,3-glucanase, two chitinases, two lipoxygenases, a basic peroxidase, a defensin, a phenylalanine-ammonia lyase, and a chalcone synthase. This study provides new insights into the interaction of soybean with *D. caulivora*, an important pathogen causing SSC, and provides information on the activation of plant defense responses.

## Introduction

Soybean [*Glycine max* (L.) Merr.] is one of the most important sources of oil and plant protein (Masuda and Goldsmith, 2009). This legume was originated in China, and later introduced in Europe and America (Qiu and Chang, 2010). At present, the major producers of soybean are United States of America (USA), Brazil, Argentina, China, and India (FAO, 2018). In addition to Brazil and Argentina, in South America, soybean is also cultivated in Uruguay, Paraguay, and Bolivia. In 2017, South America´s soybean production totaled 184.503.944 metric tons (FAO, 2018). More than 135 plant pathogens affect soybean worldwide, of which at least 30 have been reported economically important leading to significant yield losses (Vidić et al., 2013). Breeding programs for disease resistance have been established in several countries since genetic resistance, when available, is the best solution for improving global food security by reducing chemical control. Several species from the fungal genus *Diaporthe* Nitschke (asexual morph *Phomopsis*) (Sacc.) cause important diseases in soybean that affect its production, including seed decay, pod and stem blight, and stem canker (Santos et al., 2011). These diseases are commonly referred to as the *Diaporthe*/*Phomopsis* complex. *Phomopsis longicolla* (*D. longicolla*) is the primary agent of seed decay, while *Diaporthe sojae* (*D. sojae*) is the causal agent of pod and stem blight (Fernández and Hanlin, 1996; Sinclair, 1999). Soybean stem canker (SSC) is mainly caused by two different species, *Diaporthe aspalathi* (*D. aspalathi*) (syn. *Diaporthe phaseolorum* var. *meridionalis*) and *Diaporthe caulivora* (syn. *Diaporthe phaseolorum* var. *caulivora*) (Fernández et al., 1999; Pioli et al., 2003; Santos et al., 2011; Udayanga et al., 2015). SSC is one of the most widespread diseases in soybean growing regions in the world, causing in some cases losses of 100% (Backman et al., 1985). Although different fungi are associated to the abovementioned diseases, all can be isolated from infected seeds or tissues with disease (Kmetz et al., 1978; Holland and Abney, 1988; Sinclair, 1991; Sinclair, 1999). Stem canker caused by *D. caulivora* was first reported in USA in the 1970s (Backman et al., 1985). In 1992, the southern and the northern isolates causing SSC in USA were distinguished based on aggressiveness and preference in temperature, dividing the disease in two: northern stem canker (caused by *D. caulivora*) and southern canker (caused by *D. aspalathi*) (Keeling, 1982; Keeling, 1985; Keeling, 1988). The presence of *D. aspalathi* as the causal agent of SSC was reported in Argentina in 1992 (Pioli et al., 1997). *D. caulivora* was found for the first time in South America in 1999 in Argentina (Pioli et al., 2001), and in 2006, it was identified in diseased plants in Brazil (Costamilan et al., 2008). In 2002, *D. caulivora* was widely disseminated in the main soybean-producing region of Argentina, where it coexists with *D. aspalathi* (Pioli et al., 2002). In Uruguay, different isolates of *D. aspalathi* and *D. caulivora* were identified during 2012–2013 in soybean stems with canker lesions (Stewart, 2015). This was the first report of *D. aspalathi* and *D. caulivora* in Uruguay, and interestingly, 83% of the *Diaporthe* isolates causing stem canker were *D. caulivora*. The high prevalence of *D. caulivora* isolates causing SSC in Uruguay is probably due to the use of soybean genotypes, mostly from Argentinian sources, carrying resistance genes that are not effective to *D. caulivora* (Stewart, 2015). This is consistent with previous results showing that Argentinian soybean genotypes carrying Rdm1, Rdm2, Rdm3, and Rdm4 conferred resistance to *D. aspalathi* but not to *D. caulivora* (Pioli et al., 2003). Disease symptoms caused by *D. caulivora* are associated to withered brown leaves and reddish-brown discoloration and necrosis of the lower half of the stem. Yield losses by *D. caulivora* can be significant, especially when canker lesions develop early, leading to plant wilting and death in the middle of the vegetative stage (Vidić et al., 2013).

Differentiation of taxa within the *Diaporthe/Phomopsis* complex based on morphological characteristics such as pigmentation of the colony, presence or type of pycnidia (asexual state), or presence of perithecia (sexual state) is difficult since they are variable (Zhang et al., 1998; Pioli et al., 2003). The use of molecular analysis such as restriction-site variations in the internal transcribed spacers (ITS) of the nuclear ribosomal RNA genes (rDNA), and/or phylogeny inference based on the nucleotide sequence divergence in the ITS regions of the rDNA together with other genomic regions such as the translation elongation factor 1-alpha (TEF1α) gene, has been suitable to distinguish between closely related *Diaporthe* isolates that cannot be separated using morphological characteristics (Zhang et al., 1997; Santos et al., 2010; Udayanga et al., 2014; Santos et al., 2017). Polymerase chain reaction–restriction fragment length polymorphism (PCR-RFLP) analysis allows distinguishing between *D. longicolla*, *D. sojae*, *D. aspalathi*, and *D. caulivora* (Zhang et al., 1998).

Plants have developed various defense strategies to cope with invading pathogens. As part of the defense responses, pathogen-derived signals are perceived by the plant cells leading to the activation of defense genes *via* different signaling pathways. Genes encoding pathogenesis-related proteins (PR proteins) play important roles in the defense response against pathogens (van Loon et al., 2006). PR proteins accumulate at the pathogen infection sites and contribute to systemic acquired resistance (SAR) (Klessig et al., 2018). These proteins have been divided into 17 classes (PR-1–17) on the basis of their amino acid sequence identity, biological activity, or physicochemical properties (van Loon et al., 2006). Members of the PR protein family have enzymatic activities, including β-1,3-glucanase (PR-2), chitinase (PR-3, -4, -8, and -11), endoproteinase (PR-7), peroxidase (PR-9), or ribonucleases (PR-10), and have shown to exhibit either antibacterial or antifungal activity (Edreva, 2005; van Loon et al., 2006). In soybean, PR-encoding genes are induced in response to different pathogens (Moy et al., 2004; Zou et al., 2005; Upchurch and Ramirez, 2010; Schneider et al., 2011), indicating the involvement of PR proteins in soybean defense. Understanding how soybean plants defend themselves against *D. caulivora* will help to develop breeding and management strategies for resistance against this pathogen.

The focus of this study was to evaluate the diversity of *Diaporthe* species isolated from symptomatic SSC tissues, with an emphasis on *D. caulivora*, which is the main causal agent of SSC in Uruguay, and to describe *D. caulivora*–susceptible soybean plants interaction. Only few studies related to genotypic diversity of the pathogen have been performed (Pioli et al., 2003), and no information is available describing the activation of soybean defenses in response to *D. caulivora*. Here, we show that *D. caulivora* isolates with different virulence are present in Uruguay, and we describe macro- and microscopically the infection and colonization process in soybean stems. Finally, we show that plant cell walls are fortified after infection and that expression of several defense related genes are activated. Due to the agronomic importance of soybean, understanding the molecular mechanisms underlying *D. caulivora* infection and the activation of plant defenses could be useful for managing SSC in soybean plants.

## Materials and Methods

### Sampling and Isolation of Fungi

Isolates of *Diaporthe* species were recovered from canker lesions in 2012–2013 (53 isolates), and 2015 (25 isolates), taken from different plants from different regions of Uruguay, including farms in the Departments of Colonia, San José, Soriano, Flores, Treinta y Tres, Rivera, Salto, Rocha, Paysandú, Canelones, and Florida (**Supplementary Figure 1**). Symptomatic soybean stems were collected and disinfected by submerging them in 70% ethanol for 30 s. Small tissue fragments from the border of diseased stems were cut out and placed onto petri dishes containing potato dextrose agar (PDA; Difco, Detroit, USA) acidified with 0.2% lactic acid. Plates were incubated at 20°C for several days. Isolates were purified by single hyphal tip and were stored in PDA slants at 4°C.

### DNA Extraction, PCR, and Sequencing

Approximately 100 mg of mycelium was removed from 8-day-old cultures grown on PDA medium and ground with liquid nitrogen. Total DNA was extracted using the DNeasy Plant Mini Kit (Qiagen, Hilden, Germany) according to the manufacturer’s instructions. The ITS region of nuclear rDNA and a partial sequence of the TEF1α gene of each isolate was amplified using the universal fungal primers ITS4 and ITS5 (White et al., 1990), and EF1-728F and EF1-986R (Carbone and Kohn, 1999), respectively. The following PCR mix was used: 1X *Taq* buffer [20 mM (NH4)2SO4; 75 mM Tris-HCl pH = 8.8 and 0.01% (v/v) Tween 20]; 2.0 mM MgCl2; 0.2 mM of each dNTP; 1.0 µM of each primer; 2.5 U of *Taq* polymerase (Thermo Scientific); 50 ng of genomic DNA; and double-distilled water up to 50 μl. A negative control using water instead of DNA was also included. PCR cycling program for both genes consisted of an initial denaturing step for 3 min at 96°C, followed by 40 cycles of 1 min at 94°C, 1 min at 55°C, and 2 min at 72°C, and a final extension of 4 min at 72°C. The sizes of the amplified DNA products were determined by electrophoresis in a 1.5% agarose gel in Tris/acetate/EDTA (TAE) at 120 V for 30 min, stained with ethidium bromide, and visualized by UV light. PCR products were purified using the QIAquick^®^ PCR Purification Kit (Qiagen, Hilden, Germany) and sequenced in both directions at Macrogen (Korea). Obtained sequences were aligned using MEGA7 (Kumar et al., 2016), and initial species identification was done by performing Blast searches of the GenBank nucleotide database (http://www.ncbi.nlm.nih.gov). A summary of the *Diaporthe* species including information related to the name of the isolates (ID), the soybean cultivar, location, year of collection, percentage of similarity, and GenBank sequence accession numbers with the highest similarity is available in **Supplementary Table 1**. For phylogenetic analysis additional reference sequences for the different *Diaporthe* species identified were retrieved from GenBank (**Supplementary Table 2**), and two *Diaporthe vaccinii* strains were used as outgroups. In addition, sequences of *Diaporthe* isolates closely related to the identified species were added to the analysis, including *D. aspalathi*, *D. sojae*, *D. helianthi*, and *D. infecunda* (Udayanga et al., 2014; Thompson et al., 2015; Santos et al., 2017).

Phylogenetic relationships between sequences of concatenated and individual gene-trees were performed using Maximum Likelihood method based on the Kimura 2-parameter model (Kimura, 1980). The optimal model that best fit the aligned sequences data sets was determined using the JModelTest 2 program (Posada, 2008), according to Akaike Information Criterion (AICc). Bootstrap support values based on 1,000 replications were calculated for tree branches construction using the program MEGA7 (Kumar et al., 2016). Sequences were deposited in the GenBank database (MK483139–MK483213, MK507892, and MN584748–MN584826), and the corresponding access numbers are listed in **Supplementary Table 1**.

### Inter-Sequence Single Repetition–PCR Amplification

DNA extracted from 14 *D. caulivora* isolates obtained in 2015 was amplified using different inter-sequence single repetition (ISSR) primers (**Supplementary Table 3**) (Weising et al., 1989; Zhou et al., 2001; Ureña-Padilla et al., 2002; Talhinhas et al., 2005; Fan et al., 2010; Barquet et al., 2012). DNA amplification conditions for ISSR-PCR assays included an initial step of 3 min at 94°C, followed by 30 cycles of denaturation at 94°C for 30 s, annealing at primer-specific temperature for 30 s, and elongation at 72°C for 90 s. A final extension was performed at 72°C for 5 min. Each reaction contained: 1X *Taq* buffer [20 mM (NH4)2 SO4; 75 mM Tris-HCl pH 8.8 and 0.01% (v/v) Tween 20]; 2.0 mM of MgCl2, 1 μM of ISSR primer, 0.2 mM of each dNTP, 1.0 U of *Taq* polymerase (Thermo Scientific), 50 ng of genomic DNA, and double-distilled water up to 20 μl. A negative control using water instead of DNA was also included. All ISSR-PCR assays were performed twice. Amplified products were analyzed by electrophoresis on 2.0% (w/v) agarose gels in Tris/borate/EDTA (TBE) at 90 V for 50 min, stained with GelRed (Biotum, USA), and visualized by UV light (Macro VueUvis-20, Hoefer Inc, USA). Gels were scanned using a Fuji Film Starion FLA 9000 image scanner. Product sizes were estimated based on 100 bp DNA Ladder Plus (BioLabs). The obtained images were analyzed by the GelCompar II Software (Applied Maths, Brazil), where presence (1) or absence (0) of bands for each oligonucleotide primer combination used in the amplification was scored for the different isolates. Band profile reproducibility was tested by repeating the PCRs three times for the selected isolates and primers tested. A dendrogram was constructed using the unweighted pair group method with arithmetic mean (UPGMA) method (Michener and Sokal, 1957). Significance of each cluster was calculated with the cophenetic correlation (Sokal and Rohlf, 1962), which measures the correlation between the similarity measure derived from the dendrogram and the similarity matrix. The internal significance of each cluster was evaluated using the Jaccard method (Jaccard, 1901). The value used for optimization and tolerance of results was 1%.

### Morphology and Growth Examination

Morphology and growth of three selected strains were tested in five types of microbiological media, including PDA; SDA (Sabouraud dextrose agar; Britania); YPD (yeast extract–peptone–dextrose; 10 g of yeast extract, 20 g of peptone, 20 g of glucose, and 20 g of agar per liter, pH = 6.6); CMA (Corn Meal Agar; 2 g of corn meal, 15 g of agar and 1% Tween 80 per liter, pH = 6.0); and Czapeck (2 g of sodium nitrate, 0.5 g of potassium chloride, 0.5 g of magnesium sulfate, 0.01 g of iron sulfate, 1 g of phosphate bipotassium, 30 g of sucrose, and 15 g of agar per liter, pH = 6.8). Agar plugs (5 mm in diameter) from the growing edge of 8-day-old cultures grown on PDA were transferred to newly prepared media, and cultures were incubated at 24°C in 16 h light/8 h darkness photoperiod. Three replicates were established for each culture medium. Colony morphology and growth were observed and measured for 6 consecutive days.

A modified protocol was used for perithecial production (Cavinder et al., 2012). Briefly, when mycelium reached the border of the plate, the aerial mycelia was removed with a sterile toothpick, and 1 ml of Tween 60 (2.5%) was added to the surface. This procedure was repeated each time mycelium growth was evident. Development of perithecia was observed weekly, and after approximately 4 weeks perithecia were collected by scraping the surface with Tween 80 (0.1%). Asci and mature ascospores were obtained by macerating the perithecia in an electric blender (Kinematica GmbH Littau-Luzern, Switzerland) with 5 ml sterile 0.1% agar in distilled water. For nuclei staining (Hoechst 33342), asci and ascospores were hydrolyzed in 4 M HCl for 10 min according to Min et al. (2010). Confocal images were recorded on a LSM 800 laser scanning confocal microscope (Zeiss) equipped with a Zeiss Axiocam 506 color digital camera. Excitation/detection was at 405/488 nm. Length of 20 perithecium, 10 asci, and 20 ascospores were analyzed, and measurements were repeated twice from two different plates.

### Plant Material and Pathogen Inoculation

The SSC-susceptible soybean (*G. max*) cultivar Williams (PI 548631) was used for all plant assays. This cultivar is also susceptible to other soybean pathogens such as *Phakopsora pachyrhizi* (*P. pachyrhizi*) and *Phytophthora sojae* (*P. sojae*), and analysis of defense gene expression have been performed in response to biotic stress signals and *P. pachyrhizi* (Dorrance et al., 2004; Delgado-Cerrone et al., 2018; https://genevestigator.com/gv/). Three seeds were planted in a 10-cm-diameter pot filled with a mix of soil and vermiculite at a rate of 3:1. Soybean seedlings were grown in a growth room under a 16 h light/8 h dark lighting regime at 24°C. For all experiments, 3-week-old plants at V2 were used. Pathogen inoculation was performed using different methods, including the toothpick method, mycelium and ascospore inoculation, stem wounding, and detached leaf inoculation (Keeling, 1982; Ploetz and Shokes, 1985; Pioli et al., 1997; Costamilan et al., 2008; Benavidez et al., 2010; Sun et al., 2012; Campbell et al., 2017). For the toothpick inoculation method, sterile toothpicks were placed on fresh PDA plates with a PDA plug containing mycelium, and after 10 days, the toothpicks overgrown with mycelium were inserted directly into the stem under cotyledons (approx. 10 mm). A sterile toothpick was inserted into the stem as a control. For the inoculation method involving spraying of mycelium, 10 PDA plugs containing mycelium were placed in 100 ml potato dextrose broth which was agitated during 7 days in dark at 120 rpm. Mycelium was placed in approximately 30 ml of sterile distilled water and macerated in an electric blender. The resulting suspension was filtered through a 40-µm-pore-size sterile cell strainer (Falcon) to remove mycelium lumps and adjusted to optical density (DO) of 1. Soybean plants were sprayed with the corresponding suspension. For the ascospore inoculation method, perithecia were collected as mentioned previously and macerated in an electric blender with 30 ml of sterile distilled water. The resulting suspension was also filtered through a 40-µm-pore-size sterile cell strainer, and spore count was adjusted to 2 × 10^5^ per ml using a hemocytometer (Neubauer Improved Brightline, Germany). A 10 μl drop was placed onto unifoliate leaf petioles. Plants inoculated with the last three methods were placed immediately in a humid chamber for 48 h after inoculation. The stem wounding method was performed by making a thin slice along the stem with a sterile scalpel (approx. 7 mm), 1 cm above the cotyledon, and single agar plugs bearing mycelium were carefully placed on the wound. PDA plugs without pathogen were placed on wounds as a control. In both case, the wound was sealed with solid petrolatum (Vaseline). For the detached leaf inoculation, petioles with their corresponding trifoliated leaves were placed in Eppendorf vials containing half strength Murashige and Skoog medium inside petri dishes containing wet paper to avoid desiccation. Agar plugs with or without mycelium (control) were placed on midrib leaf-veins (one plug on each trifoliate leaflet), and petri dishes were sealed with parafilm. For all experiments, PDA plugs (5 mm) containing mycelium were taken from the growing edge of 5 days fresh grown cultures.

### Isolate Pathogenicity and Development of Stem Canker Symptoms

Based on molecular markers, growth, and morphology, three different isolates of *D. caulivora* (D47, D57, D08.4) were selected for pathogenicity test in plants. These tests were performed by inoculating plants using the stem wounding method as described above. Development of characteristic SSC symptoms was analyzed until death of the plant. Lesion length (mm) was determined at various time points [3, 5, 7, 11, and 14 days post-inoculation (dpi)], and observations were made to describe disease progress. Ten plants were used per treatment, and the experiment was repeated three times. Disease severity was rated in each individual stem, based on a new proposed scale. The scale includes severity values (Ni) that ranged from 1 to 7 ranked as follow: (1) plants without external symptoms; (2) stem lesions equal to or less than 15 mm; (3) stem lesions equal to or less than 25 mm; (4) stem lesions up to 50 mm in length, sometimes showing foliar symptoms; (5) stem lesions up to 75 mm long, stem girdling, typical interveinal foliar chlorosis; (6) stem lesions larger than 75 mm, plant showing interveinal foliar necrosis; and (7) dead plants (**Supplementary Figure 2**). Disease severity index was calculated using the formula: S = Σ(ni/nt × Ni), where S = severity index; ni = number of individual plants rated for severity value Ni; and nt = total number of plants per treatment (Freitas et al., 2002). The area under disease progress curve (AUDPC) was calculated according to Shaner and Finney (1977) at 14 dpi, using the formula: AUDPC = $\sum_{i=1}^{n}=[Y_{i}+Y_{i+1})/2\;*\;(X_{i+1}-X_{i})]$ where Yi = severity index according to infection index, Xi = times in days, and n = total observations number; where the final value of AUDPC is the sum of the areas by lapses, which result from the multiplication of the average reading of two consecutive dates (y value) by the lapse (days, x value) between readings. Infection index was calculated by the formula reported by Groth et al. (1999): infection index (%) = 100 *[Σ nb/(N−1)T], where n = number of individual plants in each scale value; b = scale value for each individual plant; N = 7, maximum scale rating; and T = total number of plants evaluated in each treatment. Significant differences between treatments were determined by non-parametric Kruskal–Wallis and Mann–Whitney tests using SPSS Statistics v. 21.0. The significance level for data used was *p* < 0.01.

### Visualization of Fungal Colonization and Plant Cell Wall–Associated Responses

Colonization of soybean plants by *D. caulivora* was followed by microscopic observations of stems. Control and inoculated samples were taken approximately 0.5 cm above the wound border at different times after inoculation [8, 24, 48, 72, and 96 h post-inoculation (hpi)]. Tissues were placed in a clearance solution [0.15% trichloroacetic acid (w/v) in ethanol: chloroform (4:1; v/v)] for 48 h, and the solution was changed once during this time. The samples were then washed two times for 15 min with 50% ethanol, two times for 15 min with 50mM NaOH, three times for 10 min with MilliQ water, and finally they were incubated for 30 min in 0.1 M Tris/HCl (pH = 8.5). Samples were stored in 50% (v/v) glycerol until they were cut and stained (Knight and Sutherland, 2011). Stem samples were embedded in 6% agar, and 100 μm transverse and longitudinal sections were obtained with a vibratome. Sections were maintained in 50% (v/v) glycerol until staining. Hyphae of *D. caulivora* were stained with the chitin-specific dye Wheat Germ Agglutinin–Alexa Fluor 488 conjugate (WGA-AF488, Molecular Probes, Invitrogen), and plant cell membranes and cell walls were visualized using propidium iodide (PI; Sigma) (Sun et al., 2014; Redkar et al., 2018). Samples were incubated in staining solution (20 µg/ml PI, 10 µg/ml WGA-AF488, 0.02% Tween 20 in phosphate-buffered saline, PBS) for 30 min and washed in PBS (pH = 7.4). Confocal images were recorded on an FV1000 laser scanning confocal microscope (Zeiss). Excitation/detection was at 488/500–540 nm for WGA-AF488 and at 560/600–700 nm for PI. Bright field microscopy and fluorescence microscopy were performed with an Olympus BX61 microscope (Shinjuku-ku, Tokyo, Japan), and images shown in this study were captured with MICROSUITE software package (Olympus, Tokyo, Japan) (2018). Cell wall modifications were detected with solophenyl flavine 7GFE 500 by staining the stem sections with 0.1% solophenyl flavine 7GFE 500 in water for 10 min, then rinsed in water and visualized with epifluorescence (Oliver et al., 2009). The incorporation of phenolic compounds into the plant cell walls was visualized by staining with safranin-O according to Lucena et al. (2003), and Toluidine blue according to Mellersh et al. (2002).

### Quantitative PCR

The fungal biomass in soybean stem of *D. caulivora*-infected plants at 0, 8, 24, 48, 72, and 96 hpi was quantified. Three plants per treatment were used as biological replicates. Samples were frozen in liquid nitrogen, and DNA was extracted from stem tissues (stem section of 1.5 cm including the wounded area) using the DNeasy kit (Qiagen, Hilden, Germany). DNA concentration and quality were assessed using a NanoDrop 2000 spectrophotometer (Thermo Fisher Scientific, USA). Quantitative PCR (qPCR) was performed using primers designed for the elongation factor gene of soybean (Ef1α and the β-tubulin gene of *D. caulivora* (**Supplementary Table 3**) (Miller and Huhndorf, 2005; Upchurch and Ramírez, 2010). Specificity of PCR using these species-specific primer pairs was first confirmed. qPCR was performed using the QuantiNova Probe SYBR Green PCR Kit (Qiagen, Germany) in a 96-well thermocycler (New Applied Biosystems QuantStudio 3). Each reaction consisted in 20 µl containing 10 µl of SYBR Green PCR Master mix (2X), 0.7 μM primers mix, and DNA (~25 ng). The thermocycler was programmed to run for 2 min at 95°C, followed by 40 cycles of 15 s at 94°C and 20 s at 60°C. Water was used as negative control. As a standard, a serial dilution of genomic DNA from *D. caulivora* with known concentrations (60 ng, 6 ng, 600 pg, 60 pg, and 6 pg) were analyzed to determine the sensitivity and linear range of the assay. Pathogen standard curve was generated by plotting the CT values of a 10-fold dilution series of *D. caulivora* DNA stock solution versus the logarithm of the concentration. The resulting regression equations were used to calculate fungal DNA in stem samples. Similarly, a standard curve was generated to estimate the amount of soybean DNA present in each sample. Pathogen β-tubulin estimated was expressed relative to soybean elongation factor. Each data point is the mean value of three biological replicates. Two technical replicates were used for each sample. Student’s t-test was applied to all qPCR data, and values of *p* ≤ 0.01 were considered statistically significant.

### Soybean Defense Gene Expression Analysis and Identification of Cis-Regulatory Elements

Several soybean genes were selected for expression analysis, including *PR-1* (pathogenesis-related protein-1), *PR-2* (β-1,3-glucanases), *PR-3* and *PR-4* (chitinases), *PR-10* (Ribonuclease-like protein), *LOX2* and *LOX7* (lipoxygenases), *PDF1.2* (Defensin 1.2), *IPER* (basic peroxidase), *PAL* (Phenylalanine-ammonia lyase), and *CHS* (chalcone synthase). Soybean stems inoculated with *D. caulivora* and control samples (stem section of 1.5 cm including the wounded area), were frozen in liquid nitrogen at 4, 8, 24, and 48 hpi. Samples without any treatment were also taken. Tissues were grounded with liquid nitrogen, and total RNA was extracted from 100 mg of tissues, using the RNeasy Plant Mini according to manufacturer’s instructions (Qiagen, Germany). Quality of the isolated RNA was checked by running samples on 1.2% formaldehyde agarose gel. RNA concentration was measured using a NanoDrop 2000c (Thermo Scientific, Wilmington, USA). For cDNA synthesis, 2 μg of total RNA were treated with DNase I (Thermo Scientific), and cDNA was synthesized using RevertAid Reverse transcriptase (Thermo Scientific) and oligo (dT) according to the manufacturer’s protocol. RT-qPCR was performed in a 96-well thermocycler (New Applied Biosystems QuantStudio 3) using the QuantiNova Probe SYBR Green PCR Kit (Qiagen, Germany). Each 20 µl reaction contained 10 µl of SYBR Green PCR Master mix (2X), 0.7 μM primers mix, and 1 µl of template cDNA. The thermocycler was programmed to run for 2 min at 95°C, followed by 40 cycles of 15 s at 94°C and 20 s at 60°C. Water was used as negative control. Ef1α was used as the internal control, and expression in control-treated stem tissues was used as the calibrator, with the expression level set to 1. Amplification efficiencies for different primer combinations were analyzed and all were greater than 95%. Relative expression was determined using the 2-∆∆Ct method (Livak and Schmittgen, 2001). Each data point is the mean value of three biological replicates. Two technical replicates were used for each sample. Student’s t-test was applied to all RT-qPCR data, and values of *p* ≤ 0.05 were considered statistically significant. Primers used for qPCR analyses are provided in **Supplementary Table 3**. Cis-regulatory elements/motifs were analyzed 1,000 bp upstream from the transcription start site for all soybean defense genes analyzed in this study by using Phytozome v12.1 (http://www.phytozome.net/), and Plant Cis-Acting Regulatory Elements (PlantCARE) databases, http://bioinformatics.psb.ugent.be/webtools/plantcare/html (Lescot et al., 2002).

## Results

### *Diaporthe* Isolates Obtained From SSC Lesions

**Figure 1:**
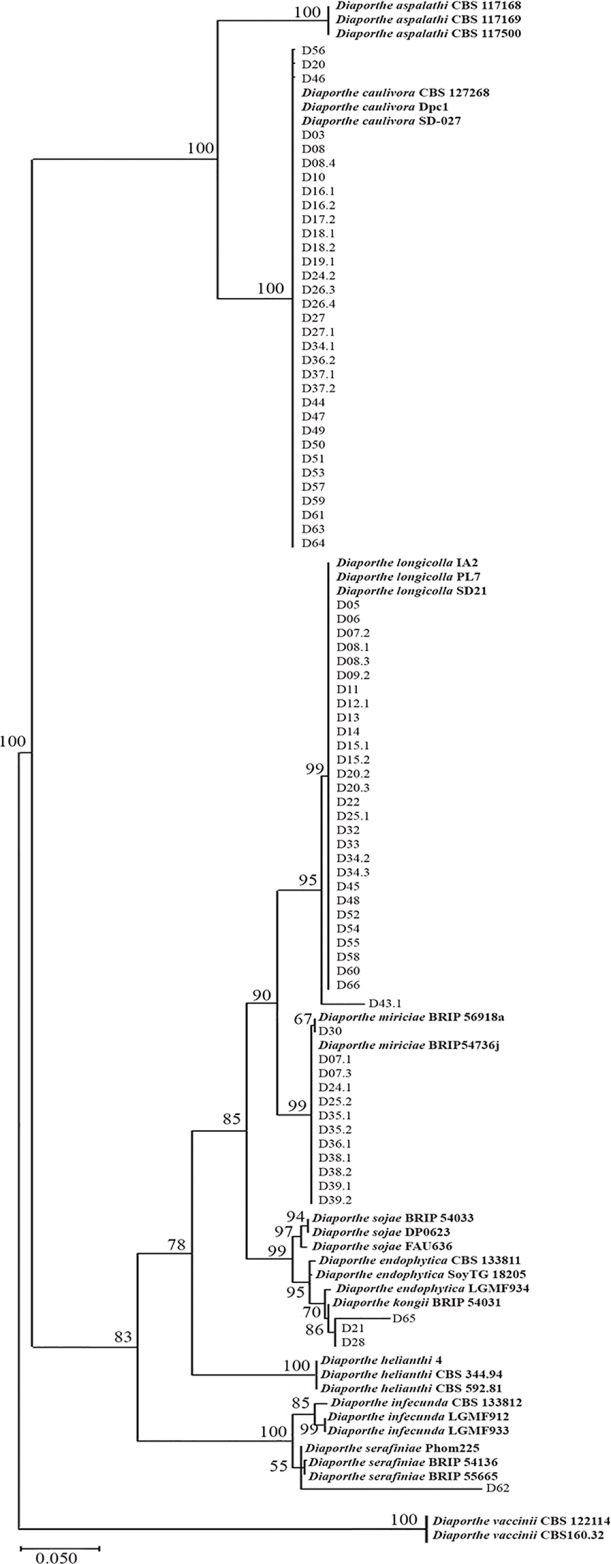
Maximum Likelihood phylogenetic tree generated from analysis of internal transcribed spacer (ITS) and translation elongation factor 1-alpha gene (TEF1α) regions of *Diaporthe* strains associated to stem canker lesions and some reference strains. The number at the branch nodes indicates bootstrap values (%) built on 1,000 replications. Ex-types, ex-epitype, or ex-isotype strains are indicated in bold face. *Diaporthe vaccinii* were used as outgroups.

Phylogenetic analysis showed that the 78 *Diaporthe* isolates recovered from SSC lesions in Uruguayan farms belong to five clades supported by high bootstrap values (**Figure 1**). The first clade consists of 33 Uruguayan isolates and the reference strains of *D. caulivora*. The second clade includes 29 Uruguayan isolates and the reference strains of *D. longicolla*. The third clade grouped 12 isolates with *D. miriciae* reference strains. The fourth clade has three isolates that grouped with strains of *D. endophytica* and *D kongii*, and the fifth clade includes one isolate that grouped with *D. serafiniae* and *D. infecunda*. The presence of a high number of *D. caulivora* isolates was consistent with previous results showing that *D. caulivora* is the main causal agent of SSC in Uruguay (Stewart, 2015). Conversely, such high number of isolates belonging to *D. longicolla* was not expected. We therefore confirmed the capability of *D. longicolla* to produce stem lesions by inoculation assays (**Supplementary Figure 3**). None of the isolates corresponded to *D. aspalathi*. Taken together, the results show that *Diaporthe* species associated to stem canker lesions in Uruguay are mainly *D. caulivora* and *D. longicolla*.

### Genetic Diversity of *D. caulivora* Based on ISSR Markers

**Figure 2:**
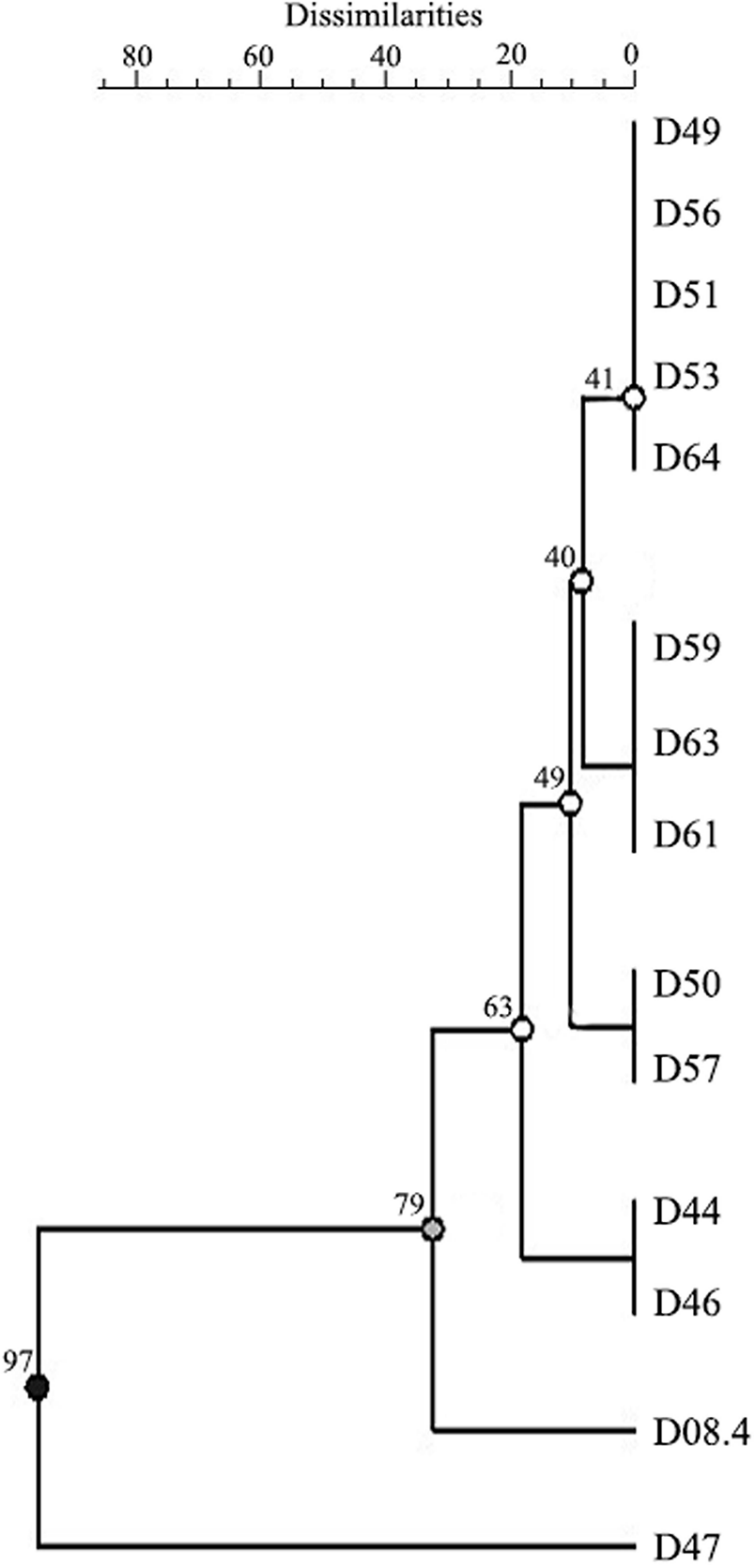
Genetic dendrogram of 14 isolates of *Diaporthe caulivora* based on three inter-sequence single repetition (ISSR) markers. Dendrogram was built using unweighted pair group method with arithmetic mean (UPGMA). Scale bar represents percentage of dissimilarities, and the branch numbers signify the cophenetic correlation value. Three clusters with two branches are distinguished according to cophenetic correlation values (branch quality is indicated in black, grey, and white).

Since few studies are available on *D. caulivora* and that it is being the most predominant species associated to SSC lesions in Uruguay, we analyzed the genetic diversity using ISSR. Primers (ACTG)4, (GTG)5, and (CAG)5 were chosen due to their informative amplification patterns, and result reproducibility (**Supplementary Figure 4**). Sixteen informative amplification bands were obtained with these ISSR primers. Although variability among *D. caulivora* was not high, differences between isolates could be distinguished in the dendrogram based on amplification patterns (**Figure 2**). Isolates were grouped in three clusters, two represented by isolate D47 and isolate D08.4, respectively, and a third cluster containing the remaining isolates. Based on these results, three *D. caulivora* isolates (D47, D57, and D08.4) were selected for further analysis.

### Morphology and Growth Characteristics of Selected *D. caulivora* Isolates

Growth characteristics of the three selected *D. caulivora* isolates were analyzed. After growing them on five different culture media, a consistent radiate growth pattern was observed, and no asexual morph was evident (**Supplementary Figure 5**). No differences in colony morphology between isolates D47, D57, and D08.4 could be detected. *D. caulivora* isolates cultured during 7 days on PDA, SDA, and YPD showed white aerial mycelia with zones of white-brownish pigmentation. On the reverse side of growth plates the colonies developed a yellow pigmentation with cream to pale brown in the center and a striate growth, especially visible on YPD. On Czapeck medium, mycelia grew translucent, rhizoid-like, without pigmentation or stomata, while mycelia cultured on CMA grew almost transparent, with zonation but without pigmentation, and growth was almost entirely inside the agar (**Supplementary Figure 5**). Colony growth was fast in PDA, SDA, and YPD, and the three *D. caulivora* isolates reached the border of the plate after 5 days (**Supplementary Figure 6**). Mycelium growth in CMA and Czapeck was slower, and only isolate D08.4 reached the border at 6 days.

**Figure 3:**
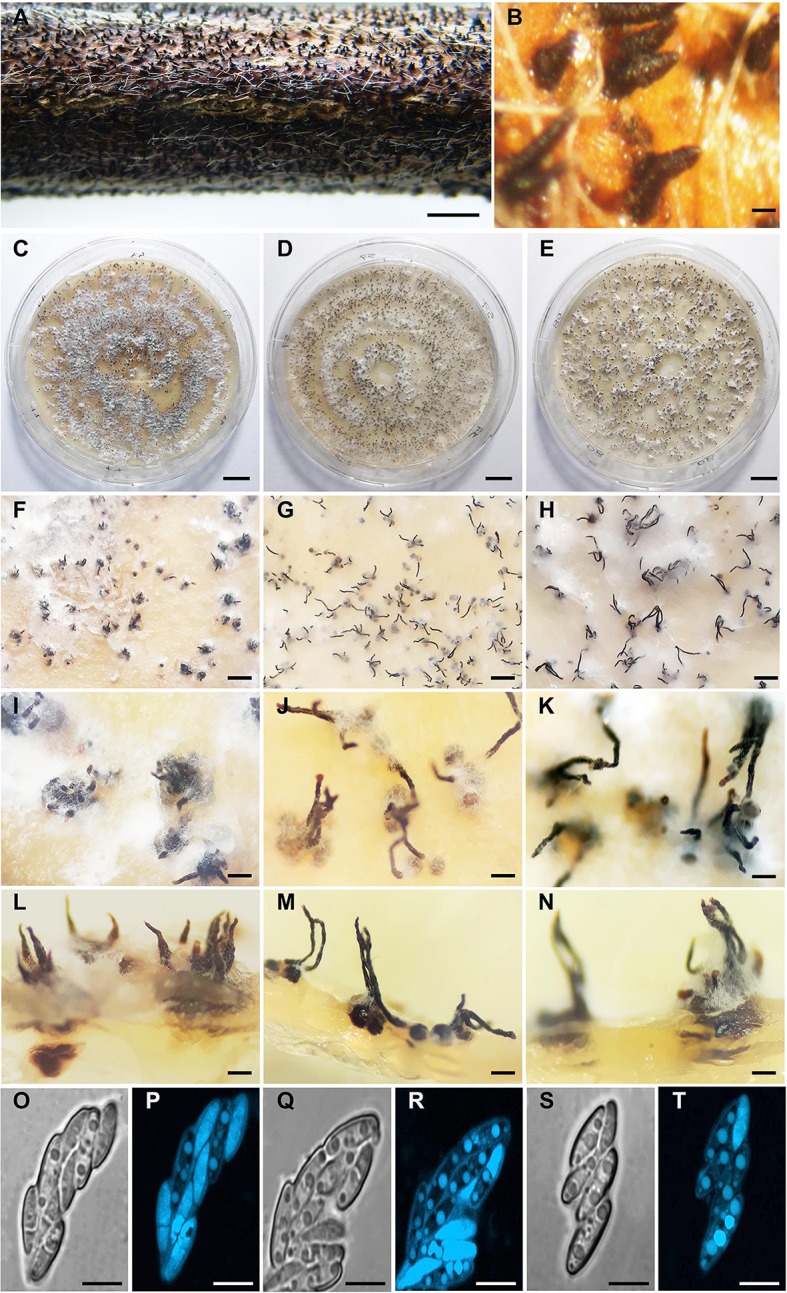
Morphology of *Diaporthe caulivora*. **(A)** Perithecia of *D. caulivora* (isolate D57) on soybean stem in water agar. **(B)** Perithecia necks of D57 protruding from soybean stem. **(C–N)** Perithecia of *D. caulivora* on potato dextrose agar (PDA) medium. **(O–T)** Asci and ascospores. Isolate D47 **(C, F, I, L, O, P)**, isolate D57 **(D, G, J, M, Q, R)**, and isolate D08.4 **(E, H, K, N, S, T).** Scale bars: **A** = 4 mm; B = 400 μm; **C–E** = 10 mm; F-H = 500 μm; **I–K** = 300 μm; **L–N** = 200 μm; **O–T** = 5 μm.

Perithecia on soybean stems were black, globose, smooth, clustered in groups, and immersed in the plant tissue (**Figures 3A, B**). Similarly, the three isolates were homothallic and showed clustered perithecia in PDA (**Figures 3C–N**). Perithecial necks were long and thin in D57 and D08.4, and short and broad in the isolate D47. Necks in D47 were 362.8 ± 27.4 µm long, 97.3 ± 10.5 µm wide at the base, and 45.5 ± 3.0 µm wide at the apex; D57 and D08.4 were 527.6 ± 32.8 and 611.1 ± 34.8 µm long, 75.1 ± 4.8 and 76.2 ± 6.3 µm wide at the base and 46.2 ± 3.7 and 46.6 ± 3.6 µm wide at the apex, respectively (**Figures 3I–N**). Asci were similar for the three *D. caulivora* isolates; unitunicate, eight-spored, oval to clavate, measuring 28.4 ± 0.7 × 5.8 ± 0.3 µm for D47; 28.8 ± 0.5 × 5.9 ± 0.3 µm for D57; and 28.8 ± 0.3 × 5.8 ± 0.2 µm for D08.4. Ascospores were two-celled, hyaline, smooth, ellipsoid to fusoid, medianly septated, slightly to non-constricted, often biguttulate (**Figures 3O–T**). Ascospore size were similar in the three *D. caulivora* isolates; 8.7 ± 0.2 × 2.1 ± 0.3 µm (D47); 8.6 ± 0.6 × 2.4 ± 0.2 µm (D57), and 9.1 ± 0.7 × 2.2 ± 0.2 µm (D08.4). Taken together, *D. caulivora* isolates were similar, but differ in having distinctive growth characteristics for D08.4 and shorter and broader perithecial necks in D47.

### Development of Stem Canker Symptoms

**Figure 4:**
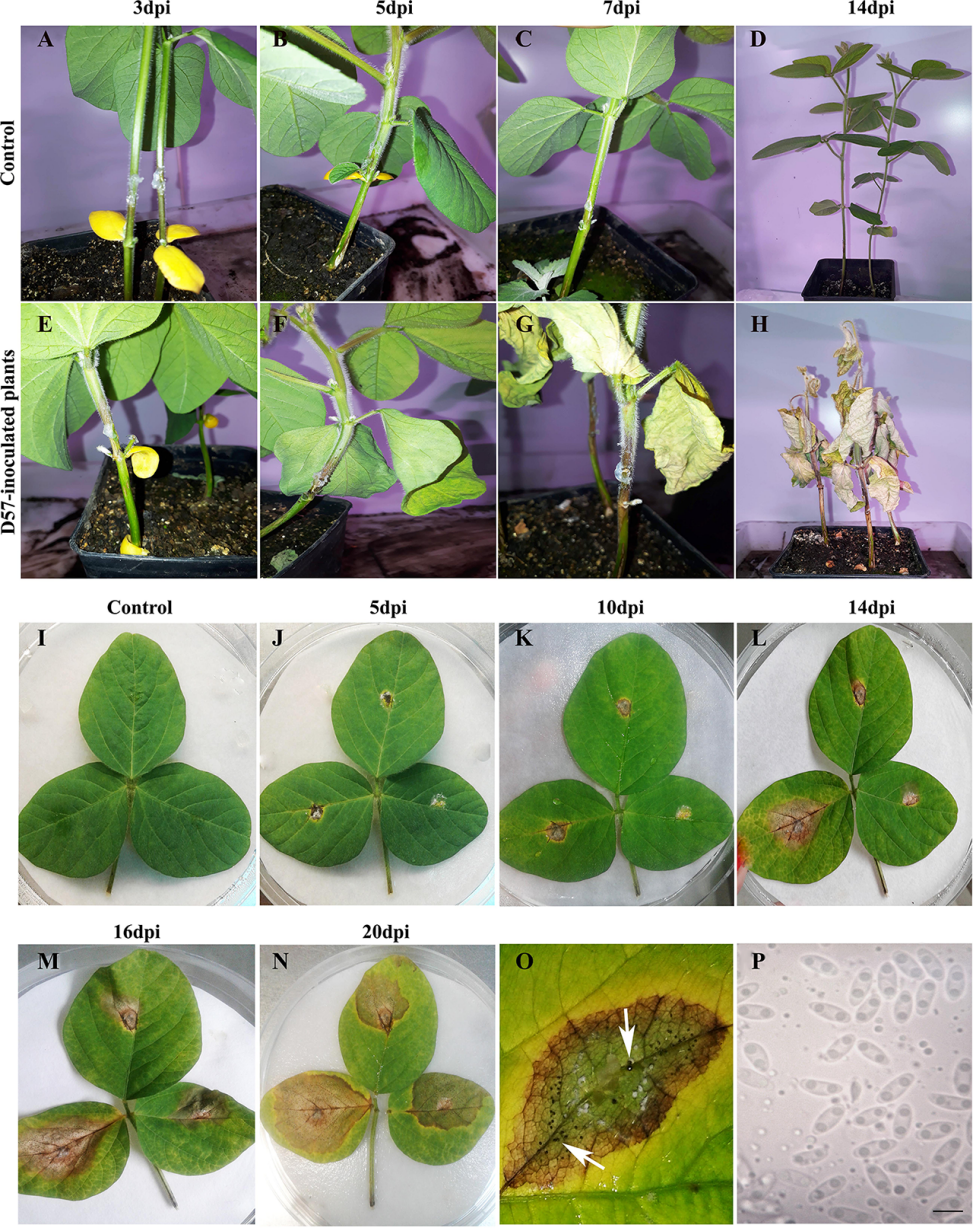
Disease symptoms in tissues infected with *Diaporthe caulivora*. Control PDA treated stems and leaves **(A–D, I)**. Development and evolution of stem canker symptoms on soybean stems **(E–H)** and leaves **(J–N)** inoculated with *D. caulivora D57* at different times post-inoculation. Pycnidia present in inoculated leaves [10 days post-inoculation (dpi)] are indicated with an arrow in **O**. Alpha conidia are shown in **P**. The scale bar in **P** represents 5 μm.

Out of the different methods used to inoculate D57, stem wounding and the application of an agar plug containing mycelium led to the development of SSC with typical brown stem lesions leading to plant decay at 14 dpi (**Supplementary Figure 7**). While with the toothpick inoculation method plants developed stem lesions, disease did not progress after 14 dpi. With the mycelium and ascospores suspension methods, only some browning of the stem was visible, and typical lesions did not form. Based on these results, the stem wounding method was selected for the rest of the work, and a more detailed analysis of disease progression was performed (**Figure 4**). Disease development was evidenced by brown discoloration of the stem and withered leaves above de canker lesion (**Figures 4E–H**). First symptoms of stem canker were observed at 3 dpi, which were more evident at 5 dpi showing typical brown lesions up to 2 mm in length (**Figures 4E, F**). Canker lesions progressed in the stems leading to leaf withering at 7 dpi, and at 14 dpi all *D. caulivora*–inoculated plants were dead (**Figures 4G, H**). Plants treated with PDA were healthy, with no apparent lesions (**Figures 4A–D**). Development of symptom in leaves was slower than in stems. Detached leaves inoculated with D57 showed necrosis and chlorosis extending from the inoculation site at 5 dpi (**Figure 4J**). No disease symptoms were visible in control leaves (**Figure 4I**). Lesions extended in the leaf tissues, and browning of veins was clearly visible (**Figures 4K–N**). In addition, pycnidia with α-conidia, which were hyaline, unicellular, ellipsoid to ovoid (6.5–8.5 × 2.0–3.1), biguttulate, and aseptate, were present in diseased tissues at 10 dpi (**Figure 4P**). When inoculated leaves were observed in more detail, areas of brown tissues were evident at the base of the trichomes (**Supplementary Figure 8**). Hyphae stained with solophenyl flavine were associated to the trichomes in leaves and stems. These results suggest that the trichomes could act as physical adhesion points facilitating fungal colonization. Additionally, after ascospores inoculation of petioles, germination occurs and hyphae developed, resulted in browning of the base of the trichomes (**Supplementary Figures 10D–F**). Browning of petioles or the base of the trichomes were not observed in control tissues (**Supplementary Figures 9A–C**).

**Figure 5:**
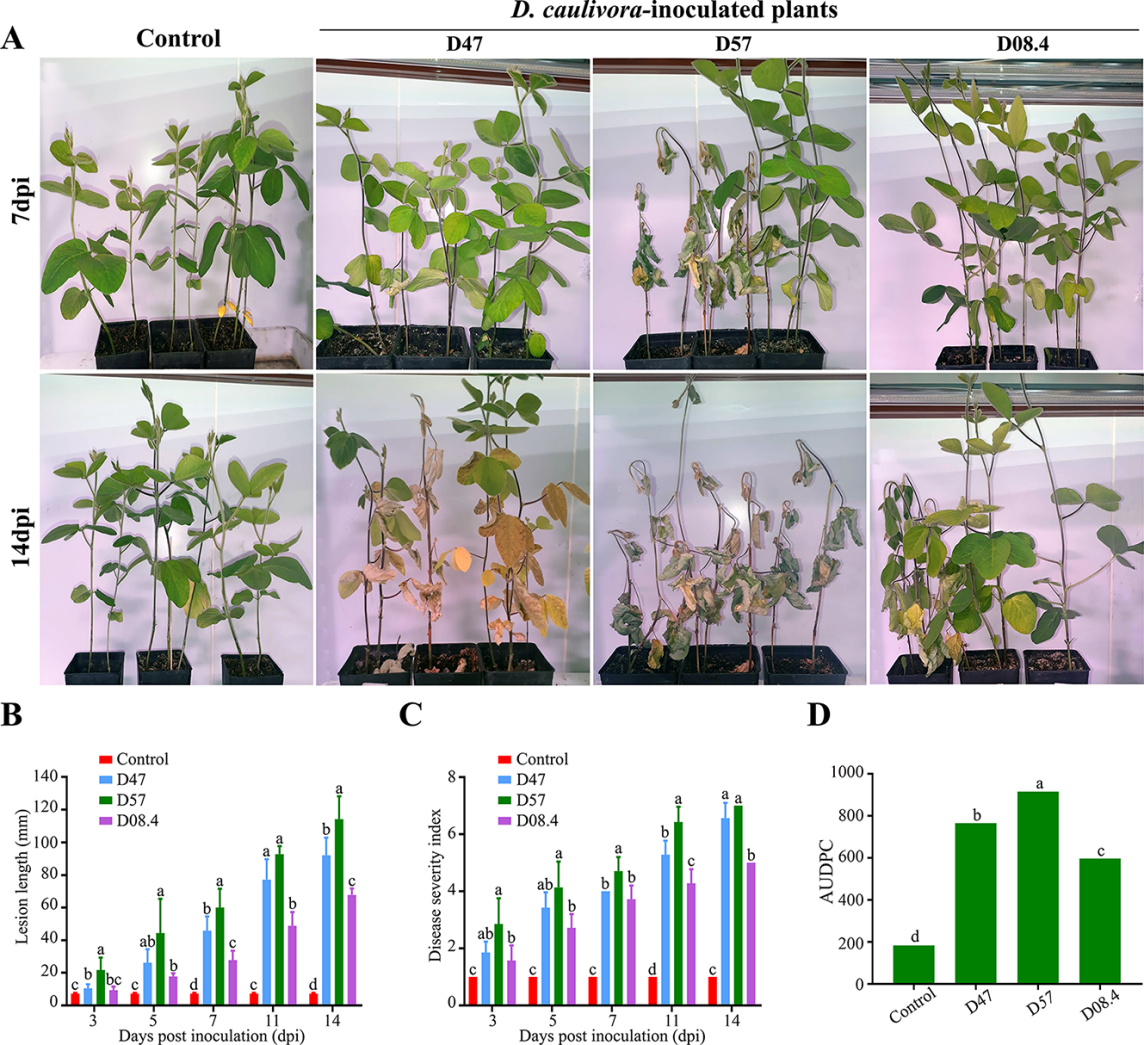
Pathogenicity assays for selected *D. caulivora* isolates. **(A)** Symptoms of susceptible plants inoculated with three *D. caulivora* isolates (D47, D57, and D08.4) at 7 and 14 dpi. **(B)** Lesion length in *D. caulivora*–inoculated stems at different dpi. **(C)** Disease severity index for *D. caulivora*–inoculated plants. **(D)** Areas under disease progress curves (AUDPC). Different letters on each time point indicate significant differences by Kruskal–Wallis and Mann–Whitney U-tests (*p* <0.01). Bars with the same letters are not significantly different. Ten plants were analyzed for each *D. caulivora* isolate. Experiments were repeated three times with similar results.

In order to evaluate the aggressiveness of the three selected *D. caulivora* isolates (D47, D57, and D08.4), plants were inoculated by stem wounding, and disease severity was visually assessed at different time points according to the proposed disease scale (**Figure 5**; **Supplementary Figure 2**). The results show that disease symptoms were more severe in plants inoculated with D57 at 7 and 14 dpi, compared to D47- and D08.4-inoculated plants (**Figure 5A**). Lesion length was significantly shorter in stems inoculated with D08.4 at 7, 11, and 14 dpi compared to D47- and D57-inoculated stems (**Figure 5B**). Significant differences in lesion length between D47- and D57-inoculated tissues were observed at 3, 7, and 14 dpi. Disease severity index increased with time, and was significantly lower at 11 and 14 dpi in soybean plants inoculated with D08.4 compared to D47 and D57 (**Figure 5C**). The AUDPC was used to compare disease progression in the *D. caulivora*–inoculated plants (**Figure 5D**). Significant differences in AUDPC values among the three *D. caulivora* isolates were observed; D08.4 = 596.7, D47 = 765.1, and D57 = 915.2. Thus, the results show that D08.4 is the less virulent isolate, while D47 and D57 are more virulent, although difference among D47 and D57 are less pronounced.

### *Diaporthe caulivora* Colonize Rapidly the Vascular Tissue

**Figure 6:**
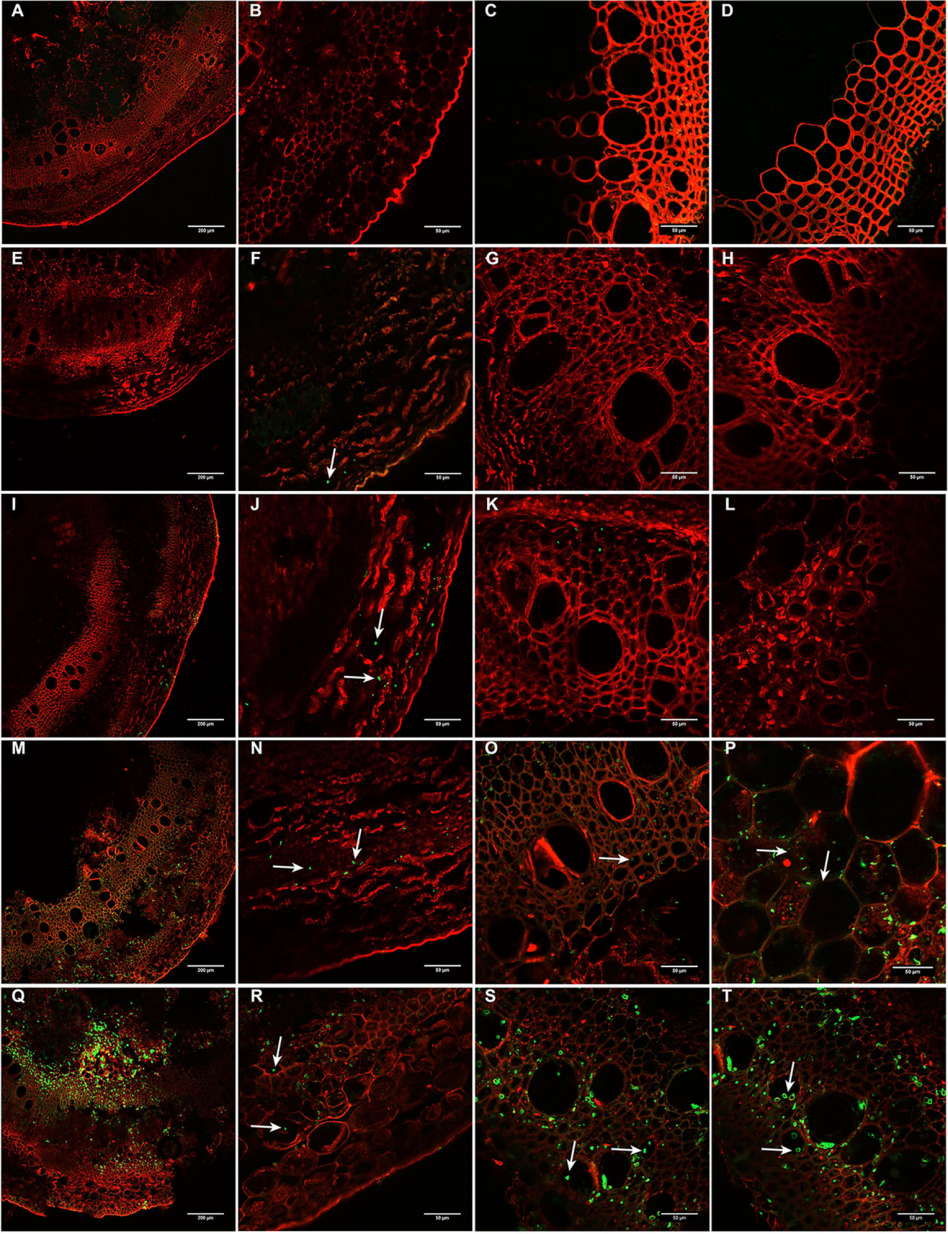
Colonization of *Diaporthe caulivora* in soybean stems at early time points. Wheat Germ Agglutinin–Alexa Fluor 488 conjugate (WGA-AF488) and propidium iodide (PI) were used for fungal cell walls and plant cell membranes/walls detection, respectively in transverse sections. **(A–D)** non-inoculated plants, **(E–H)** 24 h post-inoculation (hpi), **(I–L)** 48 hpi, **(M–P)** 72 hpi, and **(Q–T)** 96 hpi. Arrows indicate the localization of *D. caulivora* (isolate D57). Scale bars: 200 μm in **A, E, I, M, Q**, and 50 μm in **B–D, F–H, J–L, N–P, R–T**.

To gain in-depth knowledge on the infection and colonization process, inoculated stems were further evaluated at early and late time points after inoculation of soybean tissues by *D. caulivora* D57, which grouped together with most isolates. Colonization of *D. caulivora* inside the stem was monitored by confocal microscopy 8, 24, 72, and 96 hpi in transversal sections 1 cm above the wound border. WGA-AF488 was used for fungal cell walls detection, while PI detected plant cell membranes and plant cell walls (**Figure 6**). In control stem sections no fungal structures were present (**Figures 6A**–**D**). At 24 hpi, *D. caulivora* was visualized as green dots which were only detected in the cortex of the stem (**Figures 6E–H**), and at 48 hpi fungal hyphae increased in the cortex and began to colonize the phloem (**Figures 6I–L**). At 72 hpi *D. caulivora* was detected in the cortex, phloem, and tracheids and vessels of the xylem (**Figures 6M–P**). Fungal hyphae were abundant in all vascular tissues at 96 hpi (**Figures 6Q–T**). Consistently, qPCR showed that fungal biomass started to increase at 8 hpi and at 96 hpi *D. caulivora* DNA became predominant in the stem tissues (**Supplementary Figure 10**).

**Figure 7:**
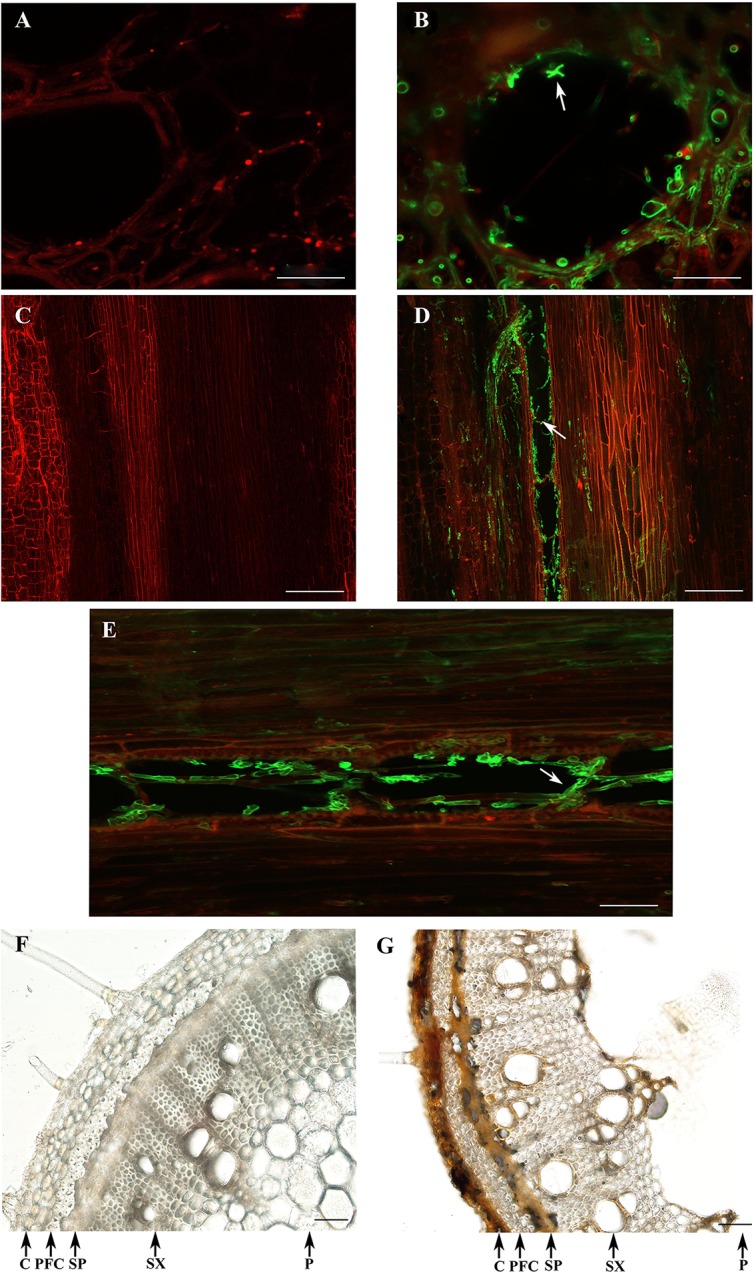
Colonization of *Diaporthe caulivora* in soybean stems at 7 dpi. Wheat Germ Agglutinin–Alexa Fluor 488 conjugate (WGA-AF488) and PI were used for fungal cell walls and plant cell membranes/walls detection, respectively in transverse **(A,B)**, and longitudinal **(C–E)** sections, and visualized with confocal microscopy. Control tissues **(A,C)** and *D. caulivora*–inoculated tissues **(B,D,E)**. Arrows indicate the localization of *D. caulivora* (isolate D57). Transversal sections of control **(F)** and *D. caulivora*–inoculated tissues **(G)**, without staining are also shown. Abbreviations: cortex (C), phloem fibers (PF), secondary phloem (SP), secondary xylem (SX), and pith (P). Scale bars: 20 μm in **A, B**; 200 μm in **C, D**; and 70 μm in **E, F, G**.

At a later time point (7 dpi), the vascular tissues were heavily colonized by *D. caulivora*, as could be observed in transverse and longitudinal sections (**Figure 7**). Fluorescence associated with the structures of the pathogen was detected towards the edges in the area comprised of vascular bundles (**Figures 7B, D, E**). Once *D. caulivora* is inside the vascular tissues, it moves throughout the stem, passing from one cell to the adjacent cell (**Figures 7D, E**). In the cross section, fluorescent points and hyphae were visible, also associated with the vascular bundles (**Figure 7B**). Differences in browning and thickness of some tissues were observed in *D. caulivora*–inoculated stems compared to control tissues (**Figures 7F, G**). Stem section of plants inoculated with *D. caulivora* showed necrosis in cortex and secondary phloem, many brown cells in the phloem fibers, and brown cell walls in the vessels. Additionally, the pith softens due to fungal infection and maceration of the tissue, and the cortex becomes smaller and less pronounced compared to control plants, probably due to changes in mechanical properties of the tissues affected by cell collapse and fungal growth. Thus, *D. caulivora* colonizes rapidly the vascular bundles leading to browning and softening of the tissues.

### *D. caulivora* Infection Activates Plant Cell Wall Reinforcement

**Figure 8:**
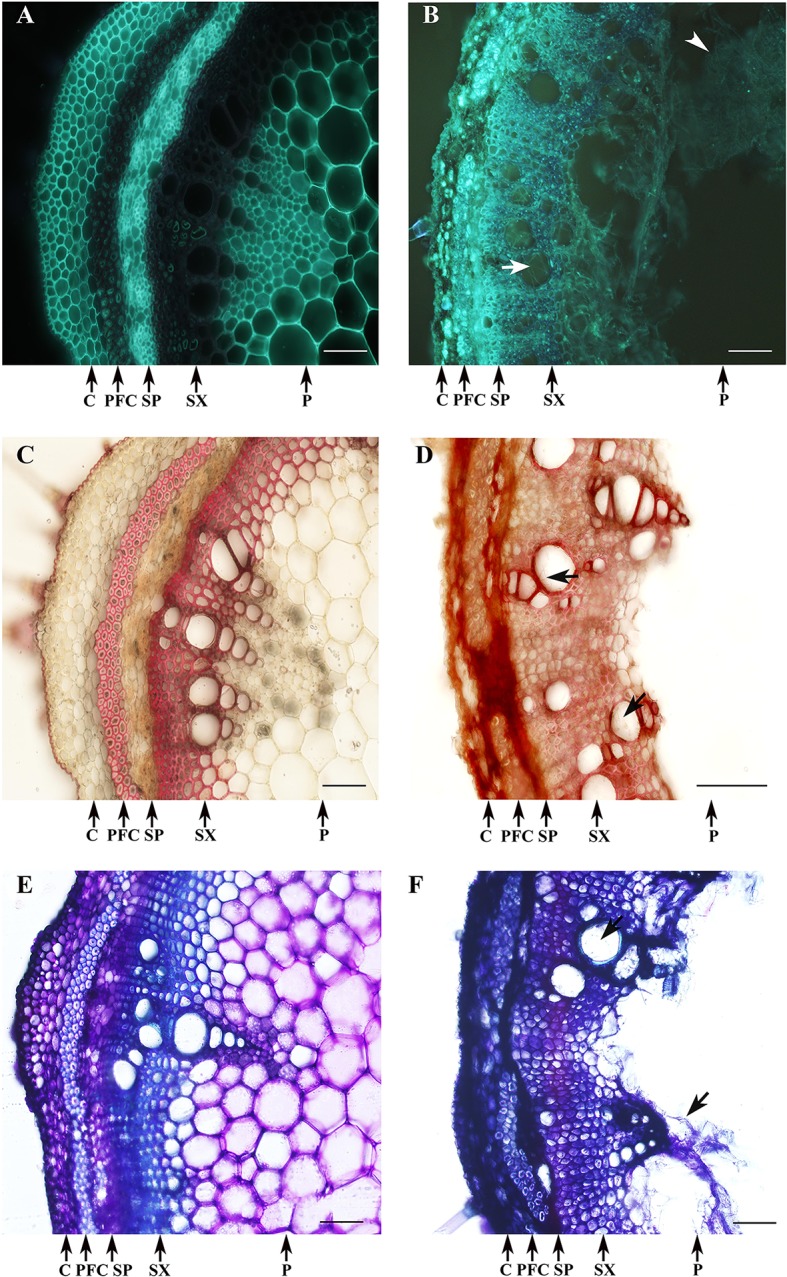
Cell wall reinforcement after *Diaporthe caulivora* colonization. Cell wall–associated defense responses in *D. caulivora*–infected tissues (isolate D57) stained at 7 dpi with solophenyl flavine **(A, B)**, safranin-O **(C, D)**, and Toluidine blue **(E, F)**. PDA-treated stem **(A,C,E)** and *D. caulivora*–infected stem **(B,D,F)**. Arrows indicates the presence of hyphae in *D. caulivora*–infected vessels. Arrowhead shows heavily infected pith tissues. Abbreviation: phloem fibers caps (PFC). Scale bars represent 70 μm.

Changes in cell wall composition were observed in *D. caulivora*–infected tissues compared to control tissues after solophenyl flavine staining. In contrast to control tissues, a bright fluorescence was detected in the cell walls of the phloem fibers and all the cells of the xylem of infected stems (**Figures 8A, B**). Hyphae inside the vessels were clearly visible at 7 dpi (**Figure 8B**). Changes in cell wall related to defense often include the incorporation of phenolic compounds into the cell walls. We therefore stained the tissue sections with safranin-O (**Figures 8C, D**). In control tissues, the phloem fibers and secondary xylem are stained, showing a pinkish-red coloration. Contrarily, all infected tissues were stained with safranin-O, including the cortex and the secondary phloem, showing an intense red-brownish coloration. Moreover, incorporation of phenolic compounds into cell walls of the vessels of the xylem of *D. caulivora*–colonized tissues was also evident by staining. Similarly, *D. caulivora*–infected cortex and secondary phloem changed in color when stained with Toluidine blue, from violet to dark blue, indicating changes in cell wall composition (**Figures 8E, F**). In addition to the observation that cell wall reinforcement occurs, these three stains evidenced that the cortex and the secondary phloem changes in *D. caulivora*–inoculated stems leading to cellular collapse.

### Defense Gene Expression in *D. caulivora*–Inoculated Soybean Stems and Cis-Acting Elements

**Figure 9:**
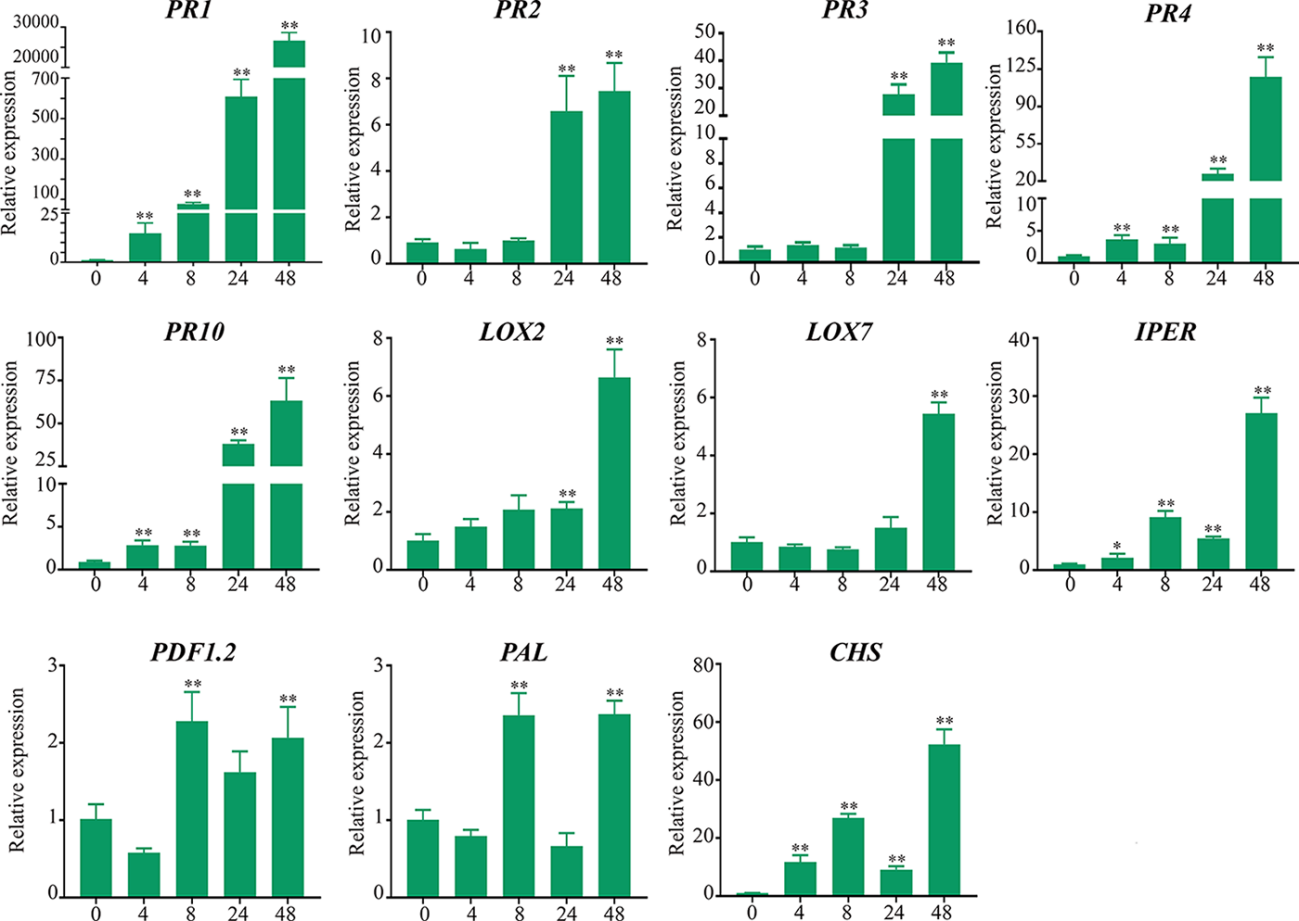
Defense-related genes expression levels in soybean stems after *Diaporthe caulivora* inoculation. RT-qPCR analysis of defense-related genes was performed at 4, 8, 24, and 48 hpi. TEF1α gene was used as the reference gene. The expression levels of PR-1, PR-2, PR-3, PR-4, PR-10; pathogenesis-related proteins; PAL, phenylalanine-ammonia lyase; CHS, chalcone synthase; LOX2, LOX7, lipoxygenases PDF-1.2, defensin 1.2 and IPER, basic peroxidase in *D. caulivora*–inoculated tissues are relative to the corresponding level of expression in control-treated tissues at the indicated time points. Results are reported as means ± SD of three biological replicates. Asterisks indicate a statistically significant difference between *D. caulivora*–inoculated (D57) and control-treated tissues (Student’s t-test, *p < 0.01; **p < 0.001). Biological significance was considered when differences in expression values were ≥ 2-fold.

Expression levels of genes encoding PR-1, PR-2 (β-1,3-glucanases), PR-3 and PR-4 (chitinases), PR-10 (Ribonuclease-like protein), LOX2 (lipoxygenase-2), LOX7 (lipoxygenase-7), PDF1.2 (Defensin 1.2), IPER (basic peroxidase), PAL (Phenylalanine-ammonia lyase), and CHS (chalcone synthase) were significantly upregulated in soybean stems inoculated with *D. caulivora* compared to control tissues (**Figure 9**). Biological significance was only considered when differences in expression values were ≥ 2-fold. Increase in transcript accumulation varied among genes and hours after *D. caulivora* inoculation. *PR-1* expression increased 14-fold after 4 hpi and continued increasing during time, reaching 26,615-fold induction at 48 hpi. *PR-4*, *PR-10*, and *IPER* showed similar expression patterns as *PR-1*, although expression levels were lower, reaching maximum of 117-, 64-, and 27-fold, respectively. *PR-2* and *PR-3* expression increased at 24 and 48 hpi compared to control tissues, reaching expression levels of 6- to 7-fold and 27- to 39-fold, respectively. *LOX2* transcript levels increased approximately 2-fold at 24 hpi, and 6-fold at 48 hpi, while *LOX7* increased only at 48 hpi (5-fold). Transcript levels of *PDF1.2* increased only 2-fold. Interestingly, both *PAL* and *CHS* have a biphasic expression profile in response to *D. caulivora*, reaching the highest expression levels at 8 and 48 hpi. Taken together, the results show that different defense genes are highly expressed in stem tissues infected with *D. caulivora*. To gain further insight into the involvement of these genes in defense responses against *D. caulivora*, the promoter regions were analyzed (**Supplementary Table 4**). Phytohormone responsive elements were present in the promoter region of the 11 defense genes analyzed, including abscisic acid (9 genes), methyl jasmonate (8 genes), ethylene (6 genes), salicylic acid (4 genes), and auxin (2 genes). These are hormones that play important roles in plant defense responses against pathogens, suggesting that they could participate in the soybean defense response against *D. caulivora*.

## Discussion

SSC is a disease caused by *Diaporthe* species that affect the production of soybean in South America and other parts of the world. SSC was responsible for soybean yield losses in several countries, including USA, Canada, Argentina, Brazil, Paraguay, Bolivia, and Italy (Backman et al., 1985; Wrather et al., 1997; Allen et al., 2017). Despite the importance of SSC, only few studies on the variability of *Diaporthe* species causing this disease have been performed, and almost no information related to the infection process and the activation of soybean defense mechanisms is available. The identification of the different species associated to SSC is important to understand the characteristics and dynamics of the disease. In this study, we have focused on Uruguayan isolates and compared the results obtained with those available for the region and other soybean-producing areas of different parts of the world. As expected from a previous study (Stewart, 2015), a high proportion of the Uruguayan *Diaporthe* isolates associated to SSC belongs to *D. caulivora* (42%), while 37% were *D. longicolla*, 15% *D. miriciae*, and 5% to *D. kongii/endophytica* and *D. serafiniae*. The high proportion of *D. longicolla* isolates associated to canker lesions was surprising, although we confirmed by inoculation assays that *D. longicolla* produces similar stem lesions as those caused by *D. caulivora*. This is consistent with recent results obtained by Ghimire et al. (2019). *D. longicolla* has been previously isolated from SSC lesions (Gebreil et al., 2015; Mathew et al., 2015); it has been associated to black zone lines on the lower stems of soybean plants (Olson et al., 2015), and it causes soybean stem blight (Cui et al., 2009). *D. longicolla* causes also *Phomopsis* seed decay leading to a reduction in seed germination of up to 90% and seed mortality (Kmetz et al., 1978; Gleason and Ferriss, 1985). *D. longicolla*, followed by *D. sojae*, are the major fungi of the *Diaporthe/Phomopsis* complex on soybean seeds, while *D. caulivora* and *D. aspalathi* have low seed-borne frequency (Kmetz et al., 1978; Zhang et al., 1999). Twelve isolates were identified as *D. miriciae*, which have also been associated with canker lesions in soybean and mung bean plants (Thompson et al., 2015). One isolate grouped with *D. serafiniae* and *D. infecunda*, and three isolates with *D. kongii* and *D. endophytica*. While *D. serafiniae* has been isolated from sunflower seeds and no reports are available describing disease symptoms in stems, *D. kongii* and *D. endophytica* have been associated to stem cankers in sunflowers in Australia (Thompson et al., 2011; Thompson et al., 2015). Although *D. kongii* and *D. endophytica* have not been correlated to SSC in field studies, *Diaporthe gulyae*, which causes *Phomopsis* stem canker of sunflower, produces reddish-brown lesions leading to plant death in soybean, suggesting that some pathogenic *Diaporthe* species from sunflower can also infect soybean and cause stem disease (Mathew et al., 2017). Consistently, *D. caulivora*, *D. longicolla*, *D. gulyae*, and *D. helianthi* cause stem canker in soybean and sunflower in inoculation assays (Mathew and Markell, 2014). Thus, since several *Diaporthe* species can cause stem lesions (Kontz et al., 2016), a better understanding of the role played by the different pathogens in the development of disease needs further investigation. Moreover, gene transfer between fungi has already been reported (Richards et al., 2011), and a non-pathogenic strains of *Fusarium oxysporum* was converted into pathogenic by the transfer of a chromosome essential for pathogenicity in tomato (Ma et al., 2010). The role played by genetic exchange between different species within the *Phomopsis/Diaporthe* complex in host specificity and pathogenicity remains to be elucidated. Interestingly, 510 potential horizontal gene transfers have been detected in the genome of *D. longicolla*, and 85.3% of them were of fungal origin (Li et al., 2017).

SSC caused by *D. caulivora* was first reported in the USA in the 1970s (Backman et al., 1985), and later it was detected in many other soybean-producing countries including Canada, Italy, the former Yugoslavia, Croatia, and Korea (Jasnić and Vidić, 1985; Zhang et al., 1997; Santos et al., 2011; Sun et al., 2012). In South America, *D. caulivora* was first found in Argentina in 1999 (Pioli et al., 2001), and in 2006 and 2012–2013, it was identified in diseased plants in Brazil and Uruguay, respectively (Costamilan et al., 2008; Stewart, 2015). In 2002, *D. caulivora* was widely disseminated in the main soybean-producing region of Argentina, where it coexists with *D. aspalathi* (Pioli et al., 2002). At present, *D. caulivora* is considered the predominant pathogen causing SSC in Argentina (Grijalba and Guillin, 2007; Grijalba and Ridao, 2012). The high number of *D. caulivora* isolates in soybean stems with canker lesions also suggests the dissemination of this pathogen in Uruguay. Consistently, no isolate corresponding to *D. aspalathi* was identified probably due to the use of resistant genotypes in Uruguayan farms (Stewart, 2015). In addition, the high presence of *D. caulivora* in the isolates analyzed could also be associated to the lower temperatures present in the southern part of Uruguay, since *D. caulivora* and *D. aspalathi* have different temperature preference (Keeling, 1988). Due to the importance of *D. caulivora* as the causal agent of SSC in Uruguay and the region, we selected three genetically different *D. caulivora* isolates (D47, D57, and D08.4) based on ISSR markers. According to growth characteristics, D08.4 could be distinguished from D47 and D57, since D08.4 grows faster in two different media under the same temperature and nutrient availability conditions, including CMA and Czapeck. Morphological characteristics were similar between the three isolates, including asci and ascospores size, which were similar to *D. caulivora* isolates from Argentina and Brazil (Pioli et al., 2001; Costamilan et al., 2008). Variations in the size of perithecial necks were observed between D47 (short), and D57 and D08.4 (long). However, it has been previously shown that this morphological feature is variable and apparently depends on moisture and light conditions (Brayford, 1990; Fernández and Hanlin, 1996). In addition, the three isolates were virulent in the susceptible genotype used, showing typical SSC symptoms. However, isolate D08.4 developed symptoms later, lesions were smaller at 7, 11, and 14 dpi, and it showed the lowest AUDPC values. D47 and D57 were more aggressive than D08.4, and based on visual symptom development and AUPDC values, D57 was the most aggressive isolate. Further studies are needed to evaluate if different races make up the Uruguayan *D. caulivora* population, aspect that could contribute to understand the epidemiology of SSC. Only few studies showing differences in pathogenicity among *D. caulivora* isolates have been performed. *D. caulivora* isolates from different origins (Argentina and USA) varied in virulence on different Argentinian susceptible genotypes (Pioli et al., 2003; Benavidez et al., 2010), suggesting that different virulence genes are present in different isolates. More information is available on the pathogenic variability among *D. aspalathi* isolates. Pioli et al. (2003) have shown the existence of variability in virulence of *D. aspalathi* isolates in Argentina, and propose the existence of four different physiological races of *D. aspalathi* based on their interaction with soybean genotypes carrying different SSC resistance genes. Similarly, occurrence of at least three races of *D. aspalathi* based on inoculation on soybean differential set has been identified in Brazil (Brumer et al., 2018). The presence of different *D. aspalathi* races can be due to selection pressure caused by deployment of specific resistant genes (Pioli et al., 1999; Pioli et al., 2003; Brumer et al., 2018). Soybean genotypes carrying Rdm1, Rdm2, Rdm3, and Rdm4 conferred resistance to *D. aspalathi* but not to *D. caulivora* (Pioli et al., 2003). Differences in susceptibility of soybean cultivars to SSC caused by *D. caulivora* have been observed previously (Pioli et al., 2003; Benavidez et al., 2010), and very recently, the first resistance gene, Rdc1, effective against *D. caulivora* was identified (Peruzzo et al., 2019). Further studies are needed to understand the mode of action of *Rdc*1, as well as identifying other resistant genes, which together with the characterization of *D. caulivora* populations could shed light to the development of effective introgression strategies in breeding programs.

*D. caulivora* colonized leaf and stem tissues, producing necrosis in the stem (canker) with brown vascular tissues extending from the inoculation site and foliar symptoms with chlorosis surrounding the necrotic areas. Pycnidia were not produced in media or inoculated stem; however, the presence of pycnidia with α conidia was observed on inoculated leaves. Development of pycnidia in *D. caulivora* is controversial. While pycnidia were not present in Argentinian *D. caulivora* isolates (Pioli et al., 2002), picnidia with α and/or β conidia were present in isolates of Unites States (Kmetz et al., 1978; Fernández and Hanlin, 1996), Korea (Sun et al., 2012), and Brazil (Brumer et al., 2018). *D. caulivora* hyphae were associated with trichomes in leaves and stems, acting probably as physical adhesion sites of the hyphae. Consistently, trichomes are preferred penetration sites for several fungi, facilitating adhesion of fungal spores and hyphae, and allowing fungal colonization progress on the tissue surface (Lazniewska et al., 2012). Ascospores on infected soybean and crop residue are the main source of inoculum (Grijalba and Ridao, 2012), and our results suggest that hyphae from germinated ascospores could be associated with trichomes. After inoculation, *D. caulivora* started to colonize the cortex at 24 hpi, progressed, and reached the phloem at 48 hpi and the xylem at 72 hpi. At 96 hpi, stem vascular tissues were heavy colonized, which was consistent with high levels of the pathogen quantitation by qPCR and development of canker lesions. Necrosis of the stem cortex was observed at 96 hpi, when symptoms were visually distinguishable. In more advanced stages of *D. caulivora* colonization necrosis of secondary phloem, browning of cells in the phloem fibers and xylem vessels were visible. In addition, changes in infected stems occur, including softening of the pith due to fungal colonization and maceration of the tissue, and the cortex and secondary phloem became smaller and less pronounced compared to control tissues. Other fungal pathogens such as *Phialophora gregata* infect soybean stems and produce similar changes in stem tissues (Impullitti and Malvick, 2014).

Plant cell wall breakdown is one of the first events involved in the infection process of fungal pathogens. These microorganisms degrade the cell walls through the combined action of a wide range of plant cell wall degrading enzymes (PCWDEs), including pectinases, pectate lyases, endopolygalacturonases, cellulases, and cutinases (Bellincampi et al., 2014). A high number of genes encoding PCWDEs have been identified in the sequenced genome of *D. longicolla*, suggesting that they could be important virulence factors (Li et al., 2017). In response to fungal infection plants induce structural defenses, including cell wall reinforcement that limits pathogen colonization of the plant tissues. Increased expression of a soybean gene encoding a protein inhibitor of fungal endopolygalacturonase (PGIP) in plants infected with *D. caulivora* (Favaron et al., 2000) suggests an active plant defense mechanism against the action of PCWDEs. Consistently, we show that *D. caulivora* colonization produces changes in the cell walls that were evidenced with solophenyl flavine staining, which detect xyloglucan (Anderson et al., 2010). Incorporation of phenolic compounds in *D. caulivora*–infected tissues was visible with safranine-O and Toluidine blue staining, suggesting reinforcement of the cell walls. Incorporation of phenolics into cell wall of xylem vessels was evident, which is consistent with the activation of a defense mechanism. Physical defenses which stop or contain the pathogen from further spread in the xylem vessels, as well as chemical defense responses that kill the pathogen or inhibit its growth, have been observed for other vascular pathogens (Yadeta and Thomma, 2013). Vascular wall coating around the infected xylem parenchyma cells and the adjacent xylem vessels has been proposed to prevent lateral and vertical spreading of vascular pathogens in the vessels (Yadeta and Thomma, 2013). Further research is needed to understand how cell wall reinforcement contributes to resistance in different soybean genotypes. RNAseq analysis of contrasting soybean genotypes together with functional studies will allow identifying genes involved in cell wall fortification mechanisms as well as other defense responses that participate in plant resistance against *D. caulivora,* leading to the development of sustainable strategies to control this disease. In addition, genome sequencing of the pathogen will provide important information on the pathogenicity mechanisms that are needed for infection as well as effector molecules that interfere with plant defense.

Activation of defense genes has been associated to basal defense against pathogens in different legumes, including soybean (Samac and Graham, 2007). Here, we observed that expression of several defense genes was upregulated in stem tissues infected with *D. caulivora* compared to control tissues. The highest transcript accumulation in *D. caulivora*–inoculated tissues compared to control tissues was observed for *PR-1*. Such high *PR-1* expression levels have been previously observed in pathogen infected *Arabidopsis* tissues (Inada et al., 2016). Genes encoding a β-1,3-glucanase (PR-2), two chitinases (PR-3 and PR-4), and a PR-10 were also highly induced after *D. caulivora* inoculation. These PRs have antimicrobial activities and have been associated to activation of defenses against different pathogen in soybean (Moy et al., 2004; Zou et al., 2005; Upchurch and Ramirez, 2010; Schneider et al., 2011). *PR1*, *PR2*, *PR3*, *PR4*, *PR5*, and *PR10* are constitutively expressed in soybean plants overexpressing NPR1 (nonexpressor of PR-1), leading to increased resistance to *P. sojae* (Fan et al., 2017). Similarly, *PR10* overexpression increases resistance against *P. sojae* (Fan et al., 2015). Here, we show that a gene encoding a basic peroxidase (IPER) is rapidly induced in stem tissues infected with *D. caulivora*, and transcript continued accumulating during fungal colonization. PR1, PR-2, and IPER are proposed to contribute to partial resistance to *P. sojae* in soybean (Vega-Sánchez et al., 2005). In addition, peroxidases and lipoxygenases (LOXs), PRs, and genes involved in the phenylpropanoid pathway are expressed in soybean tissues infected with *P. pachyrhizi* (Schneider et al., 2011). LOXs participate in the biosynthetic pathways leading to the production of different oxylipins with diverse roles in defense, including the hormone jasmonic acid (Blée, 2012). Expression levels of two *LOXs* genes are induced by *D. caulivora*, suggesting that oxylipins could play a role in defense against this fungus. *PAL* and *CHS* transcript levels also increases after *D. caulivora* inoculation. Genes encoding enzymes in the phenylpropanoid pathway, including PALs and CHSs, were also upregulated in response to *Pseudomonas syringae* and *P. sojae* in soybean (Moy et al., 2004; Zabala et al., 2006). The phenylpropanoid pathway leads to the production of compounds, which play different roles in defense, including flavonoids, coumarins, and lignans, and in soybean the reduction of flavonoids by an RNAi approach resulted in enhanced susceptibility to *P. sojae* (Subramanian et al., 2005). Expression analysis of defense genes after *D. aspalathi* infection has been performed in leaves and seeds (Upchurch and Ramirez, 2010). In response to *D. aspalathi PR-1*, *PR-2*, *PR-3*, *PR-4*, *PR10*, and *LOX7* are induced in leaves. In seeds, transcript levels of *PR-1*, *PR-2*, *CHS*, *PAL*, and *IPER* increased after *D. aspalathi* inoculation. Thus, several of these genes are important for defense responses against both *D. caulivora* and *D. aspalathi* pathogens. Interestingly, *PR-1*, *PR-5*, several *PALs*, and *CHS* expression levels are higher expressed in resistant compared to susceptible soybean genotypes after *Fusarium solani* f. sp. *glycines* infection (Iqbal et al., 2005). Similarly, transcript levels of genes encoding peroxidases and LOXs were higher in a resistant interaction of soybean with *P. pachyrhizi* compared to a susceptible interaction (Choi et al., 2008). Thus, several of these genes are important in the defense response of soybean to different pathogens, including *D. caulivora*. Phytohormones including jasmonic acid, salicylic acid, ethylene, abscisic acid, and auxin play important roles in the defense response of plants against different pathogens (Denancé et al., 2013). Here we show that all the defense genes analyzed, which are *D. caulivora*–inducible, have cis-acting regulatory element in their promoters involved in phytohormone responsiveness. Interestingly, most genes have cis-acting elements involved in abscisic acid responsiveness, and several contain elements related to methyl jasmonate, ethylene, and salicylic acid responsiveness. Further analyses are required to reveal the involvement of these hormones in the defense response against *D. caulivora*. The availability of the complete soybean genome, the presence of soybean genotypes contrasting for resistance to *D. caulivora*, and the increasing number of resources for functional genomics will help to identify key components in the plant defense response and to design strategies to enhance resistance to this important pathogen.

## Data Availability Statement

The datasets generated for this study can be found in the GeneBank database (MK483139-MK483213, MK507892, and MN584748-MN584826).

## Author Contributions

EM performed all the experiments and helped to write the manuscript. SS interpreted the data, contributed to discussions, and helped to write the manuscript. MM helped to design and to supervise the study, interpreted the data, contributed to discussions, and helped to write the manuscript. IP designed and supervised the study, interpreted the data, and wrote the manuscript. All authors read and approved the final manuscript.

## Funding

This work was supported by Agencia Nacional de Investigación e Innovación (ANII) (grant RTS-1-2014, and graduate fellowships), and Programa de Desarrollo de las Ciencias Básicas (PEDECIBA) Uruguay.

## Conflict of Interest

The authors declare that the research was conducted in the absence of any commercial or financial relationships that could be construed as a potential conflict of interest.

## References

[B1] Allen T. W. Bradley C. A. Sisson A. J. Byamukama E. Chilvers M. I. Coker C. M. (2017). Soybean yield loss estimates due to diseases in the United States and Ontario, Canada from 2010 to 2014. Plant Health Prog. 18, 19–27. 10.1094/PHP-RS-16-0066

[B2] Anderson C. T. Carroll A. Akhmetova L. Somerville C. (2010). Real-time imaging of cellulose reorientation during cell wall expansion in *Arabidopsis* roots. Plant Physiol. 152, 787–796. 10.1104/pp.109.150128 19965966 PMC2815888

[B3] Backman P. A. Weaver D. B. Morgan-Jones G. (1985). Soybean stem canker: an emerging disease problem. Plant Dis. 69, 641–647. 10.1094/PD-69-641

[B4] Barquet M. Martín V. Medina K. Pérez G. Carrau F. Gaggero C. (2012). Tandem repeat-tRNA (TRtRNA) PCR method for the molecular typing of non-*Saccharomyces* subspecies. Appl. Microbiol Biotechnol. 93, 807–814. 10.1007/s00253-011-3714-4 22113560

[B5] Bellincampi D. Cervone F. Lionetti V. (2014). Plant cell wall dynamics and wall-related susceptibility in plant-pathogen interactions. Front. Plant Sci. 5, 228. 10.3389/fpls.2014.00228 24904623 PMC4036129

[B6] Benavidez R. Pioli R. N. Morandi E. N. (2010). Response of the edamame edible soybean germplasm to *Diaporthe phaseolorum*, causal agent of soybean stem canker, in Argentina. Trop. Plant Pathol. 35, 48–51. 10.1590/S1982-56762010000100008

[B7] Blée E. (2012). Impact of phyto-oxylipins in plant defense. Trends Plant Sci. 7, 315–322. 10.1016/S1360-1385(02)02290-2 12119169

[B8] Brayford D. (1990). Variation in *Phomopsis* isolates from *Ulmus* species in the British Isles and Italy. Mycol. Res. 94, 691–697. 10.1016/S0953-7562(09)80670-9

[B9] Brumer B. B. Lopes-Caitar V. S. Chicowski A. S. Beloti J. D. Castanho F. M. Gregório da Silva D. C. (2018). Morphological and molecular characterization of *Diaporthe* (anamorph *Phomopsis*) complex and pathogenicity of *Diaporthe aspalathi* isolates causing stem canker in soybean. Eur. J. Plant Pathol. 151 (4), 1009–1025. 10.1007/s10658-018-1436-5

[B10] Campbell M. A. Li Z. Buck J. W. (2017). Development of southern stem canker disease on soybean seedlings in the greenhouse using a modified toothpick inoculation assay. Crop Prot. 100, 57–64. 10.1016/j.cropro.2017.05.026

[B11] Carbone I. Kohn L. M. (1999). A method for designing primer sets for speciation studies in filamentous ascomycetes. Mycologia 91, 553–556. 10.2307/3761358

[B12] Cavinder B. Sikhakolli U. Fellows K. M. Trail F. (2012). Sexual Development and Ascospore Discharge in *Fusarium graminearum* . J. Vis. Exp. 61, e3895. 10.3791/3895 PMC3460587 22491175

[B13] Choi J. J. Alkharouf N. W. Schneider K. T. Matthews B. F. Frederick R. D. (2008). Expression patterns in soybean resistant to *Phakopsora pachyrhizi* reveal the importance of peroxidases and lipoxygenases. Funct. Integr. Genomics 4, 341–359. 10.1007/s10142-008-0080-0 18414911

[B14] Costamilan L. M. Yorinori J. T. Almeida A. M. R. Seixas C. D. S. Binneck E. Araújo M. R. (2008). First report of *Diaporthe phaseolorum* var. *caulivora* infecting soybean plants in Brazil. Trop. Plant Pathol. 33 (5), 381–385. 10.1590/S1982-56762008000500007

[B15] Cui Y. L. Duan C. X. Wang X. M. Li H. J. Zhu Z. D. (2009). First report of *Phomopsis longicolla* causing soybean stem blight in China. Plant Pathol. 58, 799. 10.1111/j.1365-3059.2009.02057

[B16] Delgado-Cerrone L. Alvarez A. Mena E. Ponce de León I. Montesano M. (2018). Genome-wide analysis of the soybean CRK-family and transcriptional regulation by biotic stress signals triggering plant immunity. PloS One 13 (11), e0207438. 10.1371/journal.pone.0207438 30440039 PMC6237359

[B17] Denancé N. Sánchez-Vallet A. Goffner D. Molina A. (2013). Disease resistance or growth: the role of plant hormones in balancing immune responses and fitness costs. Front. Plant Sci. 4, 155. 10.3389/fpls.2013.00155 23745126 PMC3662895

[B18] Dorrance A. E. Jia H. Abney T. S. (2004). Evaluation of soybean differentials for their interaction with *Phytophthora sojae* . Plant Health Prog. 10.1094/PHP-2004-0309-01-RS

[B19] Edreva A. (2005). Pathogenesis-related proteins: research progress in the last 15 years. Gen. Appl. Plant Physiol. 31 (1-2), 105–124. 10.1.1.319.6257

[B20] Fan J. Y. Guo L. Y. Xu J. P. Luo Y. Michailides T. J. (2010). Genetic diversity of populations of *Monilinia fructicola* (Fungi, Ascomycota, *Helotiales*) from China. J. Eukaryot. Microbiol. 57, 206–212. 10.1111/j.1550-7408.2009.00467.x 20113378

[B21] Fan S. Jiang L. Wu J. Dong L. Cheng Q. Xu P. (2015). A Novel Pathogenesis-Related Class 10 Protein *Glym*4*l*, Increases Resistance upon *Phytophthora sojae* Infection in Soybean (*Glycine max* [L.] Merr.). PloS One 10 (10), e0140364. 10.1371/journal.pone.0140364 26474489 PMC4608668

[B22] Fan S. Dong L. Han D. Zhang F. Wu J. Jiang L. (2017). GmWRKY31 and GmHDL56 Enhances Resistance to *Phytophthora sojae* by Regulating Defense-Related Gene Expression in Soybean. Front. Plant Sci. 12 (8), 781. 10.3389/fpls.2017.00781 PMC5427154 28553307

[B23] FAO (2018). Food and Agriculture Organization of the United Nations. FAOSTAT Data of Crops. In: http://www.fao.org/faostat/es/#data/QC (20th April, 2019).

[B24] Favaron F. Destro T. D’Ovidio R. (2000). Transcript accumulation of polygalacturonase inhibiting protein (PGIP) following pathogen infections in soybean. J. Plant Pathol. 82 (2), 103–109. 10.4454/jpp.v82i2.1149

[B25] Fernández F. A. Hanlin R. T. (1996). Morphological and RAPD analyses of *Diaporthe phaseolorum* from soybean. Mycologia 88, 425–440. 10.2307/3760884

[B26] Fernández F. A. Philips D. V. Russin J. S. Rupe J. C. (1999). “*Diaporthe-Phomopsis* complex,” in Compendium of soybean diseases, *4th ed.* Eds. Hartman G. L. Sinclair J. B. Rupe J. C. (St. Paul, Minnesota: APS Press), 33–35.

[B27] Freitas M. A. Café Filho A. C. Nasser L. C. B. (2002). Cultural practices and genetic resistance as factors affecting soybean stem canker and plant yield in the Cerrado. Fitopatologia Bras. 27 (1), 5–11. 10.1590/S0100-41582002000100001

[B28] Gebreil A. Micijevic A. Weber A. Hyronimus L. Mathew F. (2015). Characterization of *Diaporthe* species infecting soybeans (*Glycine max* L.) in South Dakota. Proc. 100th Anniv. Meet. S. D. Acad. Sci. 94, 362 http://www.sdaos.org/wp-content/uploads/pdfs/2015/362.pdf

[B29] Ghimire K. Petrović K. Kontz B. J. Bradley C. A. Chilvers M. I. Mueller D. S. (2019). Inoculation method impacts symptom development associated with *Diaporthe aspalathi*, *D. caulivora*, and *D. longicolla* on soybean (*Glycine max*). Plant Dis. 103 (4), 677–684. 10.1094/PDIS-06-18-1078-RE103(4):677-684 30742552

[B30] Gleason M. L. Ferriss R. S. (1985). Influence of soil water potential on performance of soybean seeds infected by. Phomopsis sp. Phytopathol. 75, 1236–1241. 10.1094/Phyto-75-1236

[B31] Grijalba P. E. Guillin E. (2007). Occurrence of soybean stem canker caused by *Diaporthe phaseolorum* var. *caulivora* in the southern part of Buenos Aires province, Argentina. Australas. Plant Dis. Notes. 2, 65–66. 10.1071/DN07027

[B32] Grijalba P. Ridao Adel C. (2012). Survival of *Diaporthe phaseolorum* var. *caulivora* (causal agent of soybean stem canker) artificially inoculated in different crop residues. Trop. Plant Pathol. 37, 271–274. 10.1590/S1982-56762012000400006

[B33] Groth J. V. Ozmon E. A. Busch R. H. (1999). Repeatability and relationship of incidence and severity measures of scab of wheat caused by *Fusarium graminearum* in inoculated nurseries. Plant Dis. 83, 103–108. 10.1094/PDIS.1999.83.11.1033 30841272

[B34] Holland G. J. Abney T. S. (1988). Northern stem canker biotype of *Diaporthe phaseolorum* var. *caulivora* isolated from symptomless soybeans. Phytopathology 78, 1502.

[B35] Impullitti A. E. Malvick D. K. (2014). Anatomical response and infection of soybean during latent and pathogenic infection by Type A and B of *Phialophora gregata* . PloS One 9, e98311. 10.1371/journal.pone.0098311 24879418 PMC4039477

[B36] Inada N. Higaki T. Hasezawa S. (2016). Nuclear Function of Subclass i actin-depolymerizing factor contributes to susceptibility in arabidopsis to an adapted powdery mildew fungus. Plant Physiol. 170 (3), 1420–1434. 10.1104/pp.15.01265 26747284 PMC4775110

[B37] Iqbal M. J. Yaegashi S. Ahsan R. Shopinski K. L. Lightfoot D. A. (2005). Root response to *Fusarium solani* f. sp. *glycines*: temporal accumulation of transcripts in partially resistant and susceptible soybean. Theor. Appl. Genet. 110 (8), 1429–1438. 10.1007/s00122-005-1969-9 15815926

[B38] Jaccard P. (1901). Etude comparative de la distribution florale dans une portion des Alpes et des Jura. Bull. Soc Vaudoise Sci. Nat. 37, 547–579.

[B39] Jasnić S. M. Vidić M. (1985). Occurrence of soybean diseases in Yugoslavia. Eurosoya 3, 43–46.

[B40] Keeling B. L. (1982). A seedling test for resistance to soybean stem canker caused by *Diaporthe phaseolorum* var. caulivora. Phytopathol. 72, 807–809. 10.1094/Phyto-72-807

[B41] Keeling B. L. (1985). Soybean cultivar reactions to soybean stem canker caused by *Diaporthe phaseolorum* var. *caulivora* and pathogenic variation among isolates. Plant Dis. 69, 132–133. 10.1094/PD-69-132

[B42] Keeling B. L. (1988). Influence of temperature on growth and pathogenicity of geographic isolates of *Diaporthe phaseolorum* var. *caulivora* . Plant Dis. 72, 220–222. 10.1094/PD-72-0220

[B43] Kimura M. (1980). A simple method for estimating evolutionary rate of base substitutions through comparative studies of nucleotide sequences. J. Mol. Evol. 16, 111–120. 10.1007/BF01731581 7463489

[B44] Klessig D. F. Choi H. W. Dempsey D. A. (2018). Systemic Acquired Resistance and Salicylic Acid: Past, Present, and Future. Mol. Plant Microbe Interact. 31 (9), 871–888. 10.1094/MPMI-03-18-0067-CR 29781762

[B45] Kmetz K. T. Schmitthenner A. F. Ellett C. W. (1978). Soybean seed decay: prevalence of infection and symptom expression caused by *Phomopsis* sp., *Diaporthe phaseolorum* var. *sojae*, and *D. phaseolorum* var. *caulivora* . Phytopathology 68, 836–840. 10.1094/Phyto-68-836

[B46] Knight N. L. Sutherland M. W. (2011). A rapid differential staining technique for *Fusarium pseudograminearum* in cereal tissues during crown rot infections. Plant Pathol. 60, 1140–1143. 10.1111/j.1365-3059.2011.02462.x

[B47] Kontz B. Adhikari S. Subramanian S. Mathew F. M. (2016). Optimization and Application of a Quantitative Polymerase Chain Reaction Assay to Detect *Diaporthe* Species in Soybean Plant Tissue. Plant Dis. 100, 1669. 10.1094/PDIS-10-15-1204-RE 30686243

[B48] Kumar S. Stecher G. Tamura K. (2016). MEGA7: Molecular Evolutionary Genetics Analysis version 7.0 for bigger datasets. Mol. Biol. Evol. 33 (7), 1870–1874. 10.1093/molbev/msw054 27004904 PMC8210823

[B49] Lazniewska J. Macioszek V. K. Kononowicz A. K. (2012). Plant-fungus interface: the role of surface structures in plant resistance and susceptibility to pathogenic fungi. Physiol. Mol. Plant Pathol. 78, 24–30. 10.1016/j.pmpp.2012.01.004

[B50] Lescot M. Dehais P. Thijs G. Marchal K. Moreau Y. Van de Peer Y. (2002). PlantCARE, a database of plant cis-acting regulatory elements and a portal to tools for in silico analysis of promoter sequences. Nucleic Acids Res. 30, 325–327. 10.1093/nar/30.1.325 11752327 PMC99092

[B51] Li S. Darwish O. Alkharouf N. W. Musungu B. Matthews B. F. (2017). Analysis of the genome sequence of *Phomopsis longicolla*: a fungal pathogen causing *Phomopsis* seed decay in soybean. BMC Genomics 18 (1), 688. 10.1186/s12864-017-4075-x 28870170 PMC5584002

[B52] Livak K. J. Schmittgen T. D. (2001). Analysis of relative gene expression data using real-time quantitative PCR and the 2(-Delta Delta C(T)). Methods 25 (4), 402–408. 10.1006/meth.2001.1262 11846609

[B53] Lucena M. A. Romero-Aranda R. Mercado J. A. Cuartero J. Valpuesta V. Quesada M. A. (2003). Structural and physiological changes in the roots of tomato plants over-expressing a basic peroxidase. Physiol. Plant 118, 422–429. 10.1034/j.1399-3054.2003.00115.x

[B54] Ma L.-J. Van Der Does H. C. Borkovich K. A. Coleman J. J. Daboussi M.-J. Di Pietro A. (2010). Comparative genomics reveals mobile pathogenicity chromosomes in *Fusarium* . Nature 464, 367–373. 10.1038/nature08850 20237561 PMC3048781

[B55] Masuda T. Goldsmith P. D. (2009). World soybean production: area harvested, yield, and long-term projections. Int. Food Agribus. Manage. Rev. 12, 143–161. 10.22004/ag.econ.92573

[B56] Mathew F. M. Markell S. G. (2014)., in: Insights into the Diaporthe/Phomopsis complex infecting soybeans in the United States. Proceedings of the 24th Integrated Crop Management Conference. 10.31274/icm-180809-153

[B57] Mathew F. M. Castlebury L. A. Alananbeh K. Jordahl J. G. Taylor C. A. Meyer S. M. (2015). Identification of *Diaporthe longicolla* on dry edible pea, dry edible bean, and soybean in North Dakota. Plant Health Prog. 16, 71–72. 10.1094/PHP-RV-14-0045

[B58] Mathew F. M. Gulya T. J. Jordahl J. G. Markell S. G. (2017). First Report of Stem Disease of Soybean (Glycine max) Caused by *Diaporthe gulyae* in North Dakota. Plant Dis. 102, 240. 10.1094/PDIS-04-17-0506-PDN

[B59] Mellersh D. G. Foulds I. V. Higgins V. J. Heath M. C. (2002). H_2_O_2_ plays different roles in determining penetration failure in three diverse plant-fungal interactions. Plant J. 29, 257–268. 10.1046/j.0960-7412.2001.01215.x 11844104

[B60] Michener C. D. Sokal R. R. (1957). A quantitative approach to a problem of classification. Evolution 11, 490–499. 10.1111/j.1558-5646.1957.tb02884.x

[B61] Miller A. N. Huhndorf S. M. (2005). Multi-gene phylogenies indicate ascomal wall morphology is a better predictor of phylogenetic relationships than ascospore morphology in the *Sordariales* (Ascomycota fungi). Mol. Phylogenet. Evol. 35 (1), 60–75. 10.1016/j.ympev.2005.01.007 15737582

[B62] Min K. Lee J. Kim J. C. Kim S. G. Kim Y. H. Vogel S. (2010). A novel gene, ROA, is required for normal morphogenesis and discharge of ascospores in *Gibberella zeae* . Eukaryot Cell 9 (10), 1495–1503. 10.1128/EC.00083-10 20802018 PMC2950417

[B63] Moy P. Qutob D. Chapman B. P. Atkinson I. Gijzen M. (2004). Patterns of gene expression upon infection of soybean plants by. Phytophthora sojae. Mol. Plant Microbe Interact. 17 (10), 1051–1062. 10.1094/MPMI.2004.17.10.1051 15497398

[B64] Oliver J. P. Castro A. Gaggero C. Cascón T. Schmelz E. A. Castresana C. (2009). *Pythium* infection activates conserved plant defense responses in mosses. Planta 230 (4), 569–579. 10.1007/s00425-009-0969-4 19551405

[B65] Olson T. R. Gebreil A. Micijevic A. Bradley C. A. Wise K. A. Mueller D. S. (2015). Association of *Diaporthe longicolla* with black zone lines on mature soybean plants. Plant Health Prog. 16 (3), 118–122. 10.1094/PHP-RS-15-0020

[B66] Peruzzo A. M. Hernández F. E. Pratta G. R. Ploper L. D. Pioli R. N. (2019). Identification and inheritance of an Rdc gene resistance to soybean stem canker (*Diaporthe phaseolorum* var. *caulivora*). Eur. J. Plant Pathol. 154, 1179–1184. 10.1007/s10658-019-01716-z

[B67] Pioli R. N. Gattuso S. Prado D. Borghi A. (1997). Recent outbreak of stem canker (*D. phaseolorum* var. *meridionalis*) of soybean (*Glycine max*) in Santa Fe Argentina. Plant Dis. Note 81 (10), 1215. 10.1094/PDIS.1997.81.10.1215A 30861718

[B68] Pioli R. N. Morandi E. N. Gosparini C. O. Borghi A. L. (1999). First report on pathogenic variability of different isolates of *Diaporthe phaseolorum* var. *meridionalis* on soybean in Argentina. Plant Dis. 83, 1071. 10.1094/PDIS.1999.83.11.1071B 30841283

[B69] Pioli R. N. Morandi E. N. Bisaro V. (2001). First report of soybean stem canker caused by *Diaporthe phaseolorum* var. *caulivora* in Argentina. Plant Dis. 85, 95. 10.1094/PDIS.2001.85.1.95B 30832081

[B70] Pioli R. N. Morandi E. N. Luque A. Gosparini C. O. (2002). Recent Outbreak of Soybean Stem Canker Caused by *Diaporthe phaseolorum* var. *caulivora* in the Main Soybean-Producing Region of Argentina. Plant Dis. 86, 1403. 10.1094/PDIS.2002.86.12.1403A 30818452

[B71] Pioli R. N. Morandi E. N. Martínez M. C. Lucca F. Tozzini A. Bisaro V. (2003). Morphologic, molecular, and pathogenic characterization of *Diaphorte phaseolorum* variabillity in the core soybean-producing area of Argentina. Phytopathology 93 (2), 136–146. 10.1094/PHYTO.2003.93.2.136 18943127

[B72] Ploetz R. C. Shokes F. M. (1985). Soybean stem canker incited by ascospores and conidia of the fungus causing the disease in the southeastern United States. Plant Dis. 69 (11), 990–992. 10.1094/PD-69-990

[B73] Posada D. (2008). jModelTest: phylogenetic model averaging. Mol. Biol. Evol. 25 (7), 1253–1256. 10.1093/molbev/msn083 18397919

[B74] Qiu L. J. Chang R. Z. (2010). “The origin and history of soybean,” in The soybean: botany, production and uses. Eds. Singh G. (Oxford, UK: CABI Publishing).

[B75] Redkar A. Jaeger E. Doehlemann G. (2018). Visualization of Growth and Morphology of Fungal Hyphae in planta using WGA-AF488 and Propidium Iodide Co-staining. Bio-protocol Bio 101, 2942. 10.21769/BioProtoc.2942

[B76] Richards T. A. Leonard G. Soanes D. M. Talbot N. J. (2011). Gene transfer into the fungi. Fungal Bio. Rev. 25 (2), 98–110. 10.1016/j.fbr.2011.04.003

[B77] Samac D. A. Graham M. A. (2007). Recent Advances in Legume-Microbe Interactions: Recognition, Defense Response, and Symbiosis from a Genomic Perspective. Plant Physiol. 144 (2), 582–587. 10.1104/pp.107.096503 17556521 PMC1914196

[B78] Santos J. M. Correia V. G. Phillips A. J. L. (2010). Primers for mating-type diagnosis in *Diaporthe* and *Phomopsis*: their use in teleomorph induction *in vitro* and biological species definition. Fungal Biol. 114 (2-3), 255–270. 10.1016/j.funbio.2010.01.007 20943136

[B79] Santos J. M. Vrandečić K. Cosić J. Duvnjak T. Phillips A. J. (2011). Resolving the *Diaporthe* species occurring on soybean in Croatia. Persoonia 27, 9–19. 10.3767/003158511X603719 22403474 PMC3251324

[B80] Santos L. Alves A. Alves R. (2017). Evaluating multi-locus phylogenies for species boundaries determination in the genus *Diaporthe* . PeerJ 5, e3120. 10.7717/peerj.3120 28367371 PMC5372842

[B81] Schneider K. T. van de Mortel M. Bancroft T. J. Braun E. Nettleton D. Nelson R. T. (2011). Biphasic gene ex-pression changes elicited by *Phakopsora pachyrhizi in* soybean correlate with fungal penetration and haustoria formation. Plant Physiol. 157, 355–371. 10.1104/pp.111.181149 21791600 PMC3165884

[B82] Shaner G. Finney R. E. (1977). The effect of nitrogen fertilization on the expression of show-mildewing resistance in Knox wheat. Phytopathology 67, 1051–1056. 10.1094/Phyto-67-1051 40934422

[B83] Sinclair J. B. (1991). Latent infection of soybean plants and seeds by fungi. Plant Dis. 75, 220–224. 10.1094/PD-75-0220

[B84] Sinclair J. B. (1999). “ *Diaporthe/Phomopsis* Complex,” in Compendium of Soybean Diseases, 4th ed., vol. 31 (St. Paul, MN: The American Phytopathological Society).

[B85] Sokal R. Rohlf J. (1962). The comparison of dendrograms by objective methods. Taxon 11, 33–40. 10.2307/1217208

[B86] Stewart S. (2015). Caracterización del agente causal del cancro del tallo de la soja en Uruguay. Agrociencia Uruguay 19 (1), 69–76. 10.2477/vol19iss1pp69-76

[B87] Subramanian S. Graham M. Y. Yu O. Graham T. L. (2005). RNA interference of soybean isoflavone synthase genes leads to silencing in tissues distal to the transformation site and to enhanced susceptibility to *Phytophthora sojae* . Plant Physiol. 13, 1345–1353. 10.1104/pp.104.057257 PMC1088325 15778457

[B88] Sun S. Van K. Kim M. Y. Min K. H. Lee Y. W. Lee S. H. (2012). *Diaporthe phaseolorum* var. *caulivora*, a causal agent for both stem canker and seed decay on soybean. Plant Pathol. J. 28, 55–59. 10.5423/PPJ.NT.10.2011.0194

[B89] Sun H. Hu X. Ma J. Hettenhausen C. Wang L. Sun G. (2014). Requirement of ABA signalling-mediated stomatal closure for resistance of wild tobacco to *Alternaria alternata* . Plant Pathol. 63, 1070–1077. 10.1111/ppa.12181

[B90] Talhinhas P. Sreenivasaprasad S. Neves-Martins J. Oliveira H. (2005). Molecular and phenotypic analyses reveal association of diverse *Colletotrichum acutatum* groups and a low level of *C. gloeosporioides* with olive anthracnose. Appl. Environ. Microbiol. 71 (6), 2987–2998. 10.1128/AEM.71.6.2987-2998.2005 15932994 PMC1151867

[B91] Thompson S. M. Tan Y. P. Young A. J. Neate S. M. Aitken E. A. B. Shivas R. G. (2011). Stem cankers on sunflower (*Helianthus annuus*) in Australia reveal a complex of pathogenic *Diaporthe (Phomopsis)* species. Persoonia 27, 80–89. 10.3767/003158511X617110 22403478 PMC3251322

[B92] Thompson S. M. Tan Y. P. Shivas R. G. Neate S. M. Morin L. Bissett A. (2015). Green and brown bridges between weeds and crops reveal novel *Diaporthe* species in Australia. Persoonia 35, 39–49. 10.3767/003158515X687506 26823627 PMC4713110

[B93] Udayanga D. Castlebury L. A. Rossman A. Y. Chukeatirote E. Hyde K. D. (2014). Insights into the genus *Diaporthe*: phylogenetic species delimitation in the *D. eres* species complex. Fungal Diversity. 67 (1), 203–229. 10.1007/s13225-014-0297-2

[B94] Udayanga D. Castlebury L. A. Rossman A. Y. Chukeatirote E. Hyde K. D. (2015). The *Diaporthe* sojae species complex: phylogenetic re-assessment of pathogens associated with soybean, cucurbits and other field crops. Fungal Biol. 119 (5), 383–407. 10.1016/j.funbio.2014.10.009 25937066

[B95] Upchurch R. G. Ramirez M. E. (2010). Defense-related gene expression in soybean leaves and seeds inoculated with *Cercospora kikuchii* and *Diaporthe phaseolorum* var. meridionalis. Physiol. Mol. Plant Pathol. 75, 64–70. 10.1016/j.pmpp.2010.08.007

[B96] Ureña-Padilla A. R. Mackenzie S. J. Bowen B. W. Legard D. E. (2002). Etiology and population genetics of *Colletotrichum* spp. causing crown and fruit rot of strawberry. Phytopathology 92 (11), 1245–1252. 10.1094/PHYTO.2002.92.11.1245 18944251

[B97] van Loon L. C. Rep M. Pieterse C. M. (2006). Significance of inducible defense-related proteins in infected plants. Annu. Rev. Phytopathol. 44, 135–162. 10.1146/annurev.phyto.44.070505.143425 16602946

[B98] Vega-Sánchez M. E. Redinbaugh M. G. Costanzo S. Dorrance A. E. (2005). Spatial and temporal expression analysis of defense-related genes in soybean cultivars with different levels of partial resistance to *Phytophthora sojae*. Physiol. Mol. Plant Pathol. 66, 175–182. 10.1016/j.pmpp.2005.07.001

[B99] Vidić M. Dordević V. Petrović K. Miladinović J. (2013). Review of soybean resistance to pathogens. Ratarstvo i povrtarstvo 50 (2), 52–61. 10.5937/ratpov50-4038

[B100] Weising K. Weigand F. Driesel A. J. Kahl G. Zischer H. Epplen J. T. (1989). Polymorphic simple GATA/GACA repeats in plant genomes. Nucleic Acids Res. 17 (23), 10128. 10.1093/nar/17.23.10128 2602131 PMC335264

[B101] White T. J. Bruns T. Lee S. Taylor J. W. (1990). “Amplification and direct sequencing of fungal ribosomal RNA genes for phylogenetics,” in PCR Protocols: a guide to methods and applications. Eds. Innis M. A. Gelfand D. H. Sninsky J. J. White T.J. (New York: Academic Press), 315–322.

[B102] Wrather J. A. Anderson T. R. Arsyad D. M. Gai J. Ploper L. D. Porta-Puglia A. (1997). Soybean disease loss estimates for the top 10 soybean producing countries in 1994. Plant Dis. 81, 107–110. 10.1094/PDIS.1997.81.1.107 30870925

[B103] Yadeta K. A. Thomma B. P. (2013). The xylem as battleground for plant hosts and vascular wilt pathogens. Front. Plant Sci. 4, 97. 10.3389/fpls.2013.00097 23630534 PMC3632776

[B104] Zabala G. Zou J. Tuteja J. Gonzales D. O. Clough S. J. Vodkin L. O. (2006). Transcriptome changes in the phenylpropanoid pathway of *Glycine max* in response to *Pseudomonas syringae* infection. BMC Plant Biol. 6, 26. 10.1186/1471-2229-6-26 17083738 PMC1636052

[B105] Zhang A. W. Hartman G. L. Riccioni L. Chen W. D. Ma R. Z. Pedersen W. L. (1997). Using PCR to distinguish *Diaporthe phaseolorum* and *Phomopsis longicolla* from other soybean fungal pathogens and to detect them in soybean tissues. Plant Dis. 81, 1143–1149. 10.1094/PDIS.1997.81.10.1143 30861709

[B106] Zhang A. W. Riccioni L. Pedersen W. L. Kollipara K. P. Hartman G. L. (1998). Molecular identification and phylogenetic grouping of *Diaporthe phaseolorum* and *Phomopsis longicolla* isolates from soybean. Phytopathology 88, 1306–1314. 10.1094/PHYTO.1998.88.12.1306 18944833

[B107] Zhang A. W. Hartman G. L. Curio-Penny B. Pedersen W. L. Becker K. B. (1999). Molecular Detection of *Diaporthe phaseolorum* and *Phomopsis longicolla* from Soybean Seeds. Phytopathology 89 (9), 796–804. 10.1094/PHYTO.1999.89.9.796 18944708

[B108] Zhou S. Smith D. R. Stanosz G. R. (2001). Differentiation of *Botryosphaeria* species and related anamorphic fungi using inter simple or short sequence repeat (ISSR) fingerprinting. Mycol. Res. 105 (8), 919–926. 10.1016/S0953-7562(08)61947-4

[B109] Zou J. Rodriguez-Zas S. Aldea M. Li M. Zhu J. Gonzalez D. O. (2005). Expression profiling soybean response to *Pseudomonas syringae* reveals new defense-related genes and rapid HR-specific down regulation of photosynthesis. Mol. Plant Microbe Interact. 18, 1161–1174. 10.1094/MPMI-18-1161 16353551

