# Supplementary figures and images for: Soybean Stem Canker Caused by Diaporthe caulivora; Pathogen Diversity, Colonization Process, and Plant Defense Activation
Source: Front Plant Sci. 2020 Jan 23;10:1733. doi: 10.3389/fpls.2019.01733 (PMC7011206; doi:10.3389/fpls.2019.01733)

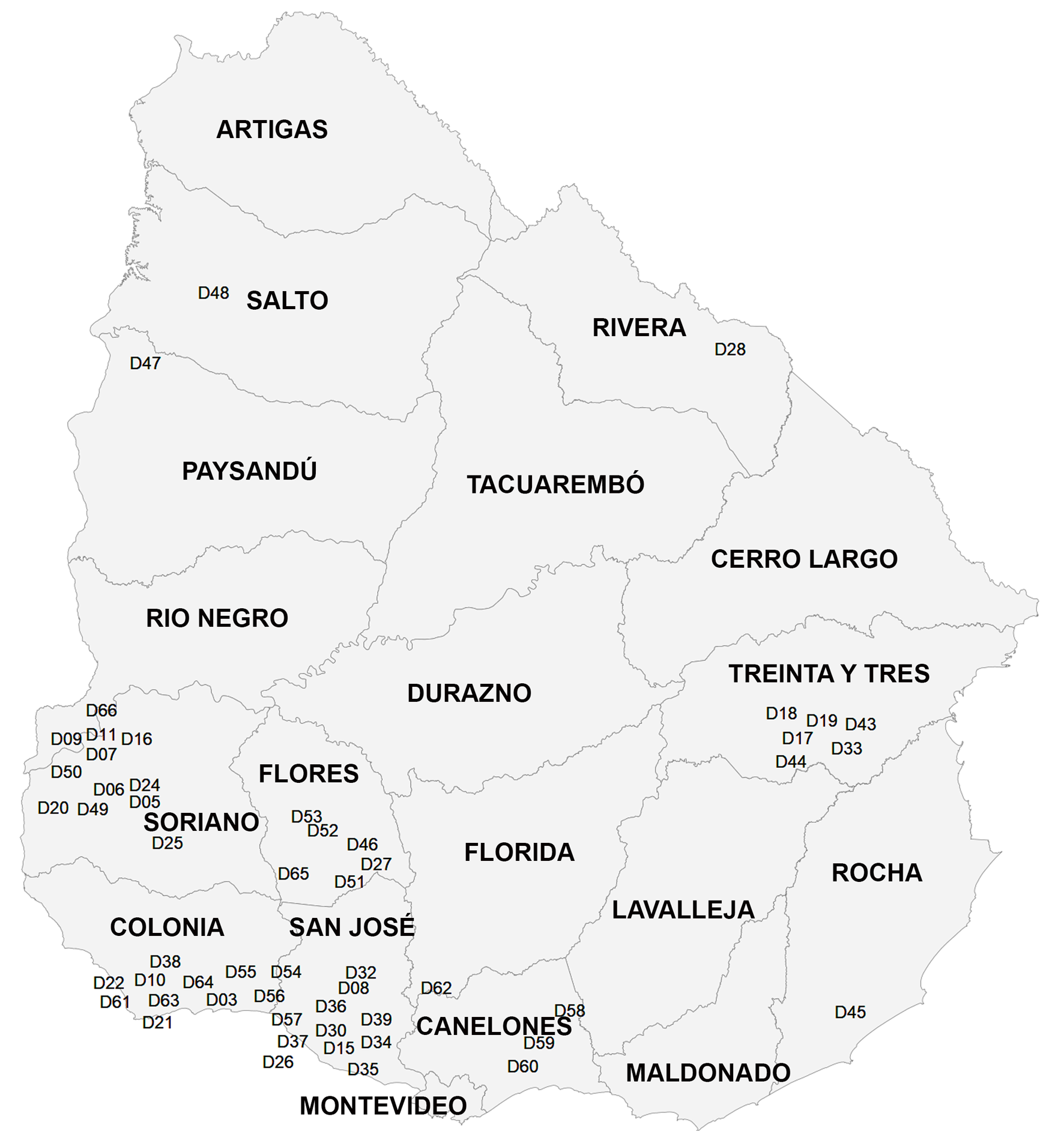

Supplement: Supplementary file 1 [file Image_1.tif]

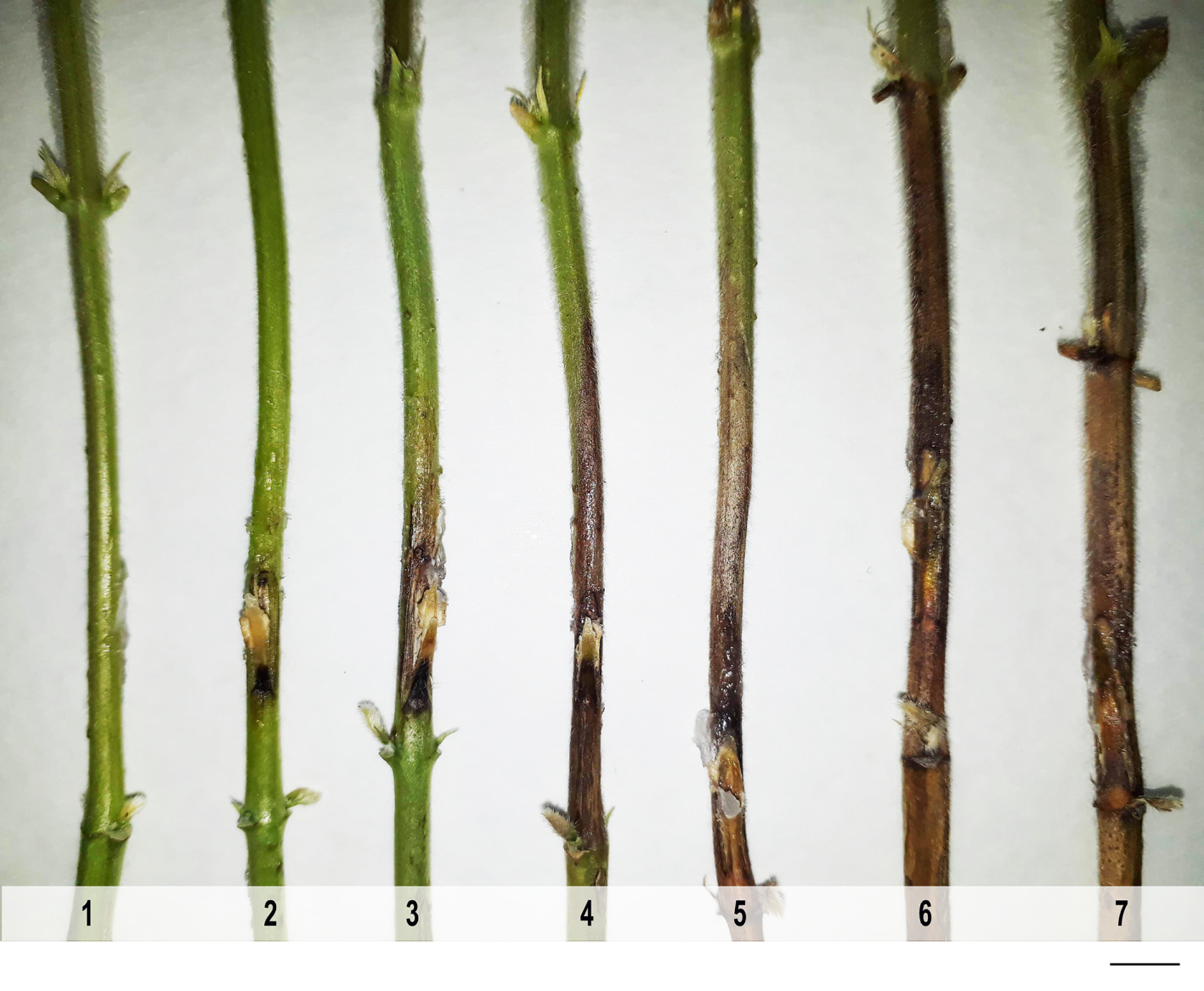

Supplement: Supplementary file 2 [file Image_2.tif]

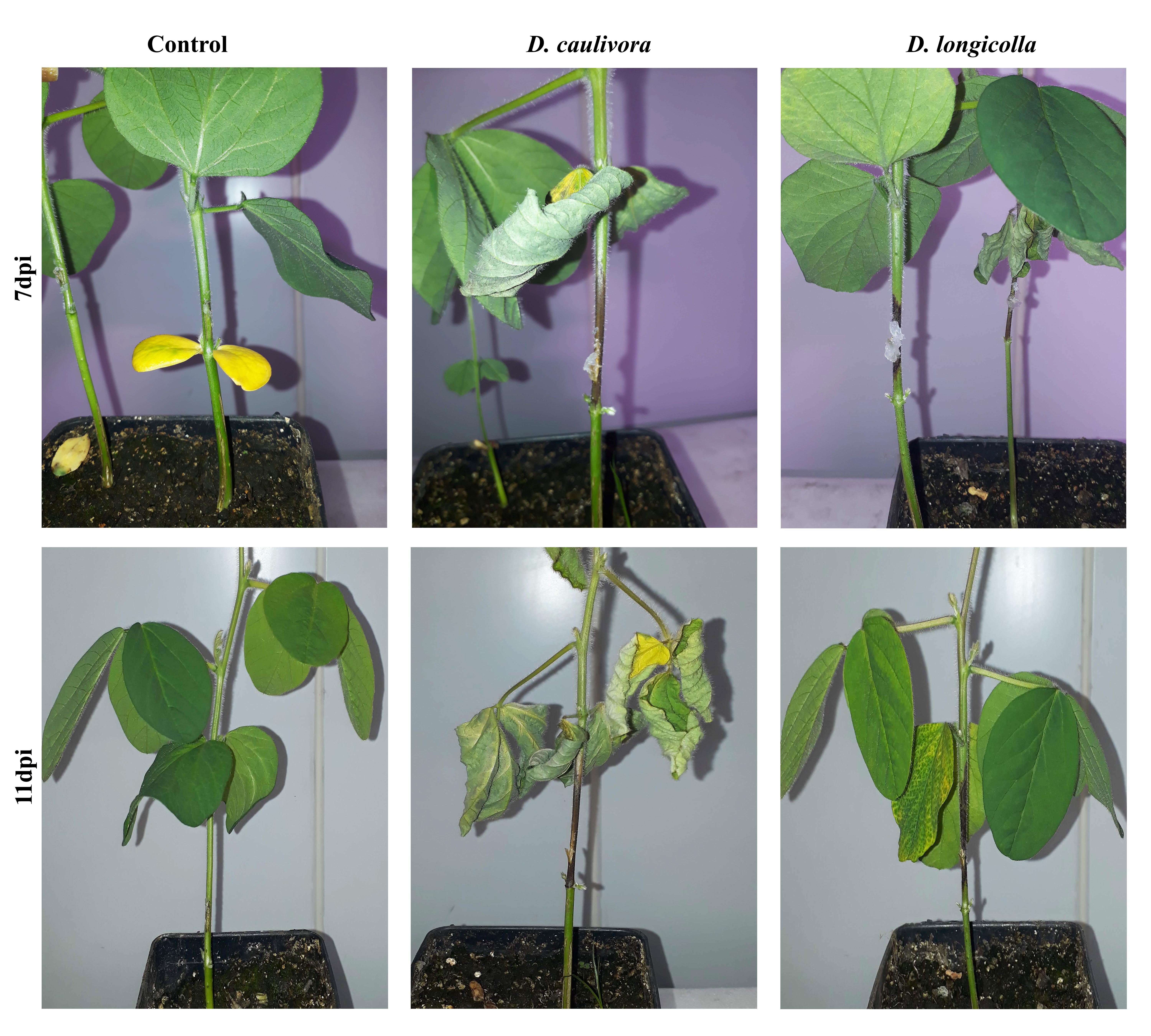

Supplement: Supplementary file 3 [file Image_3.jpeg]

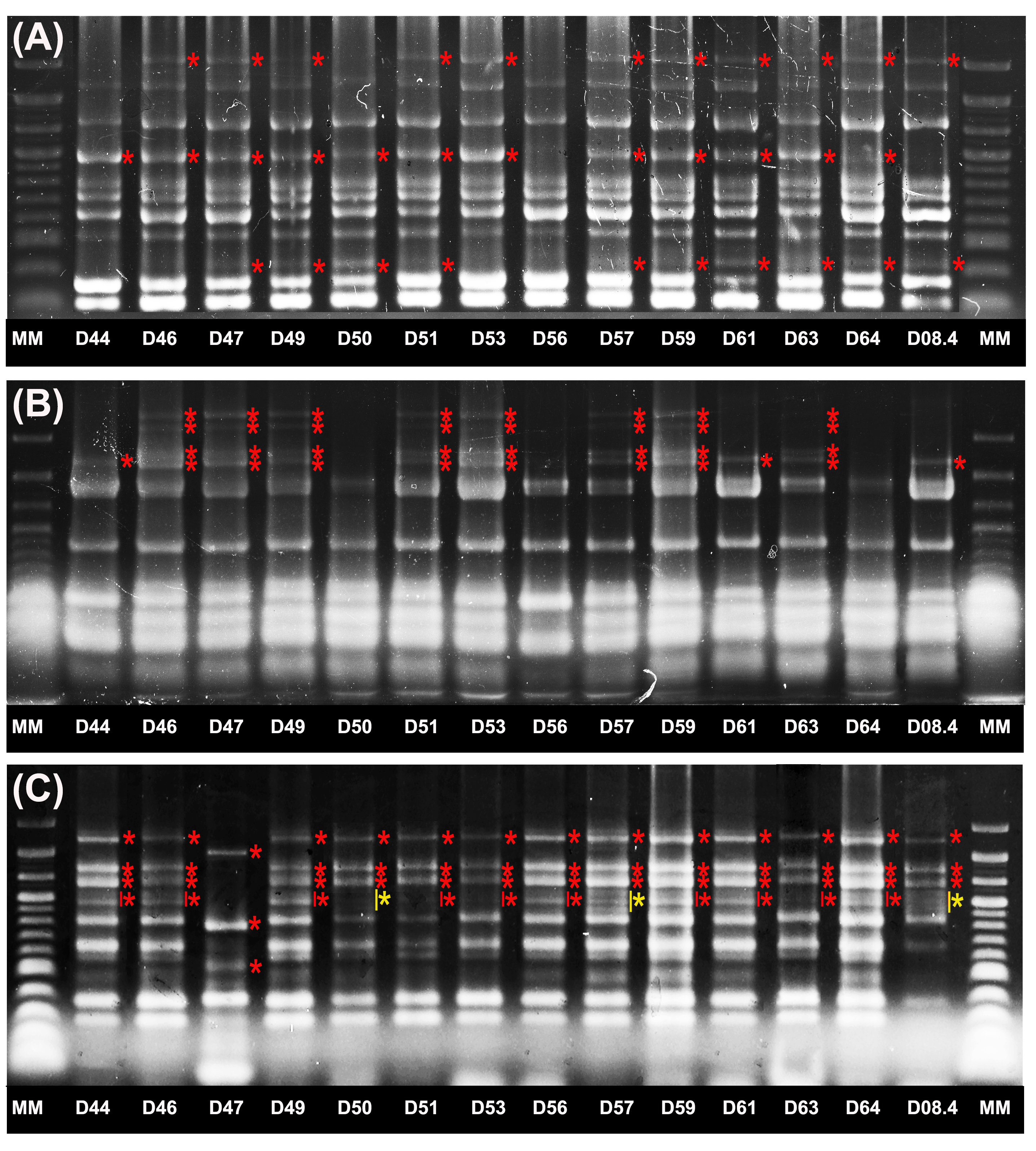

Supplement: Supplementary file 4 [file Image_4.tif]

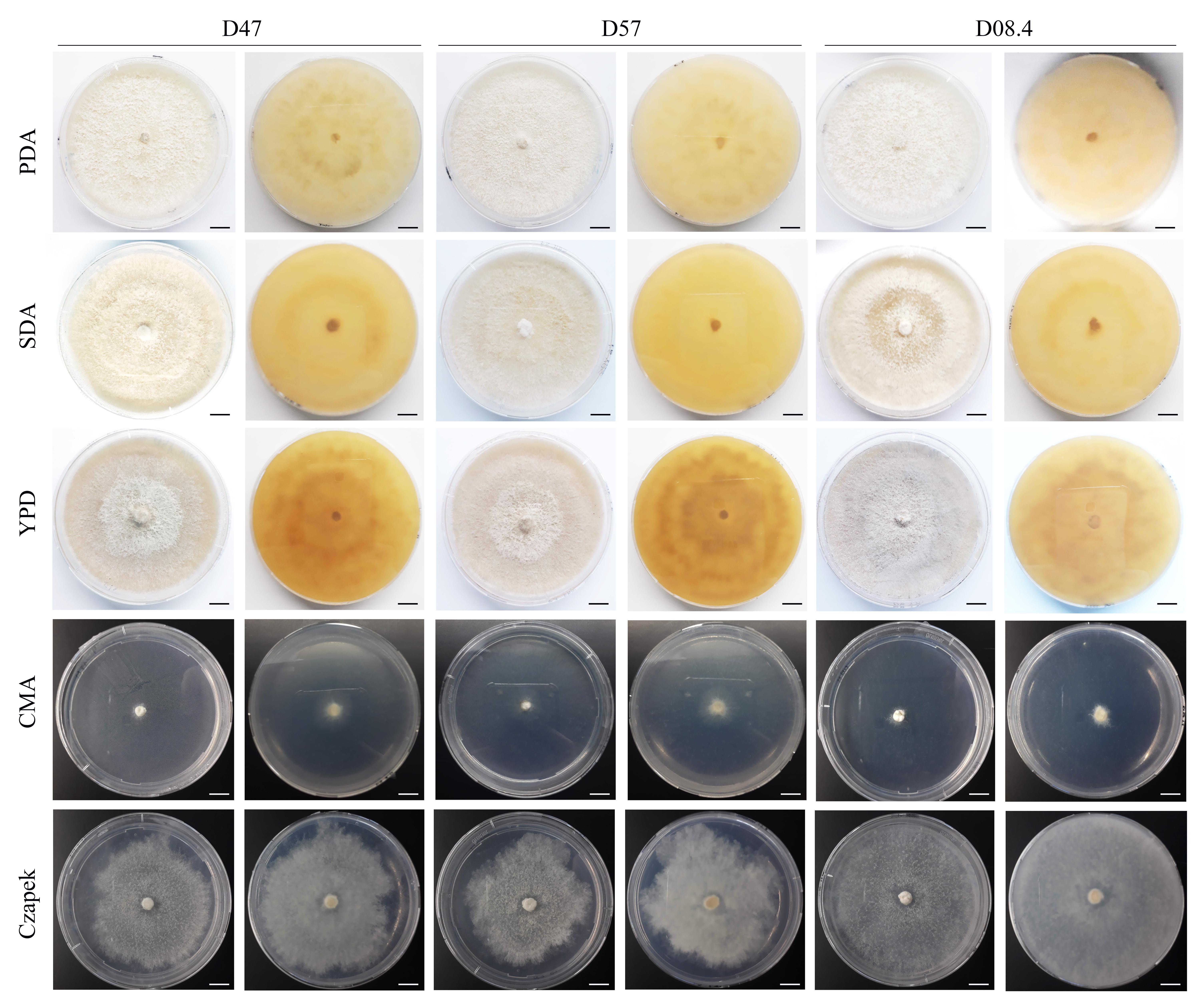

Supplement: Supplementary file 5 [file Image_5.jpeg]

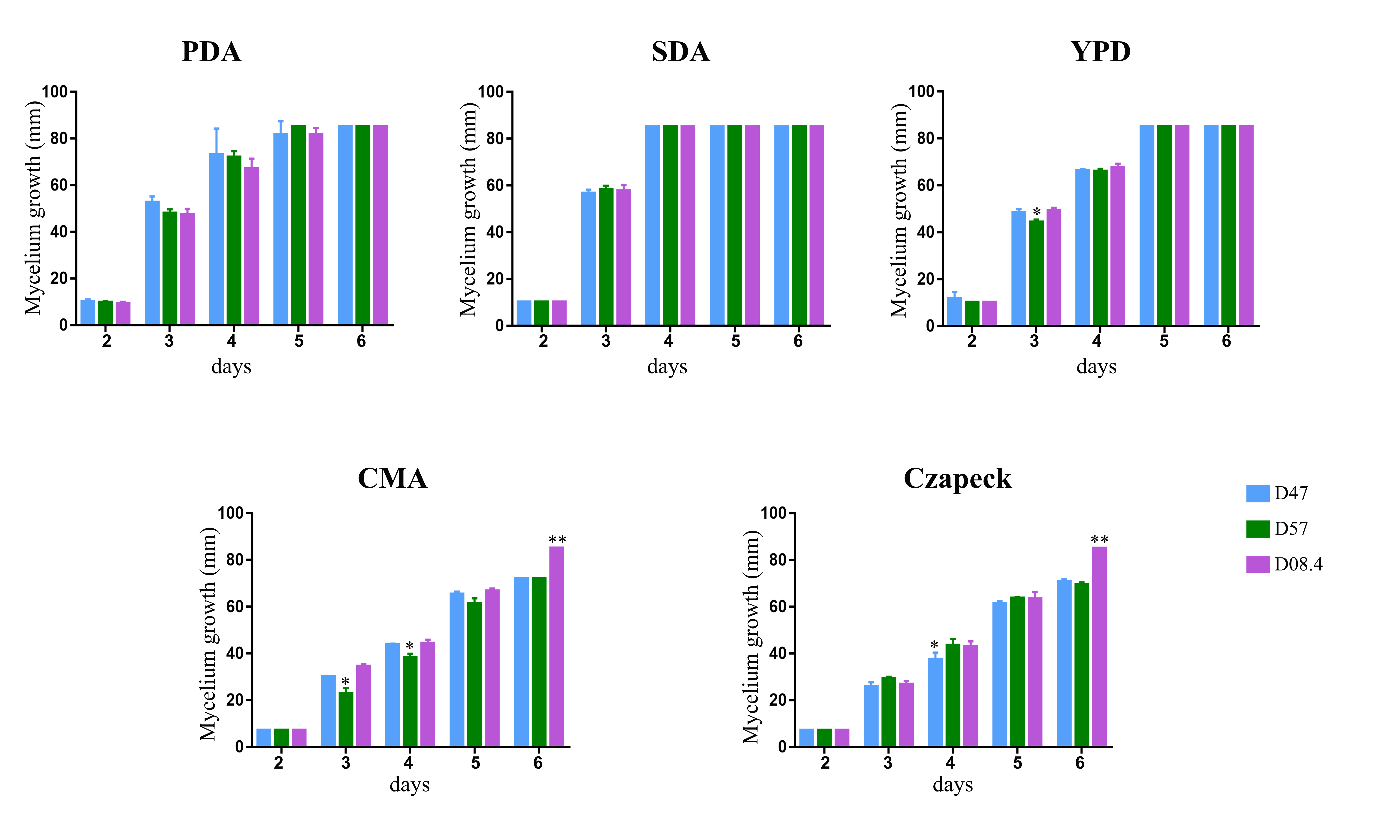

Supplement: Supplementary file 6 [file Image_6.tif]

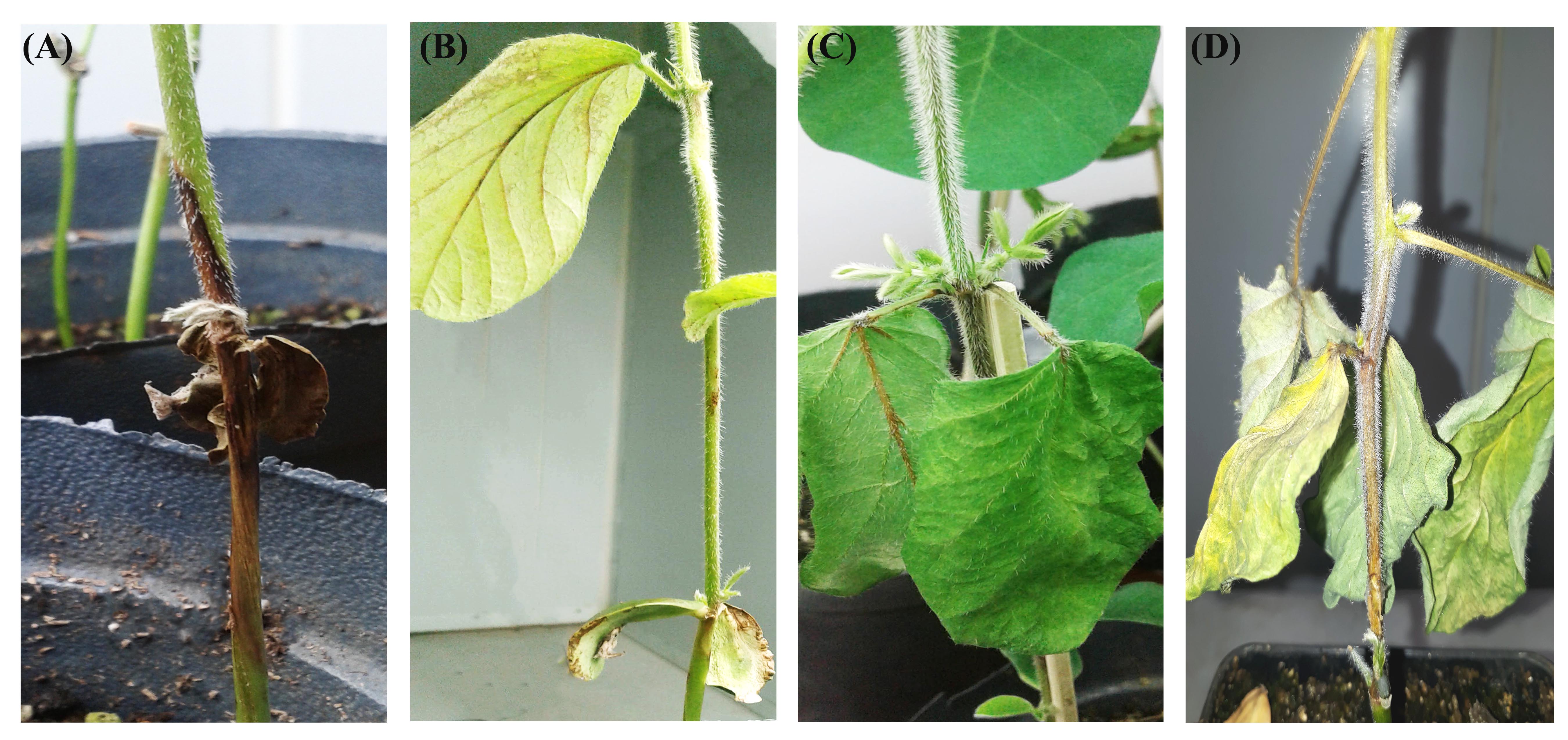

Supplement: Supplementary file 7 [file Image_7.jpeg]

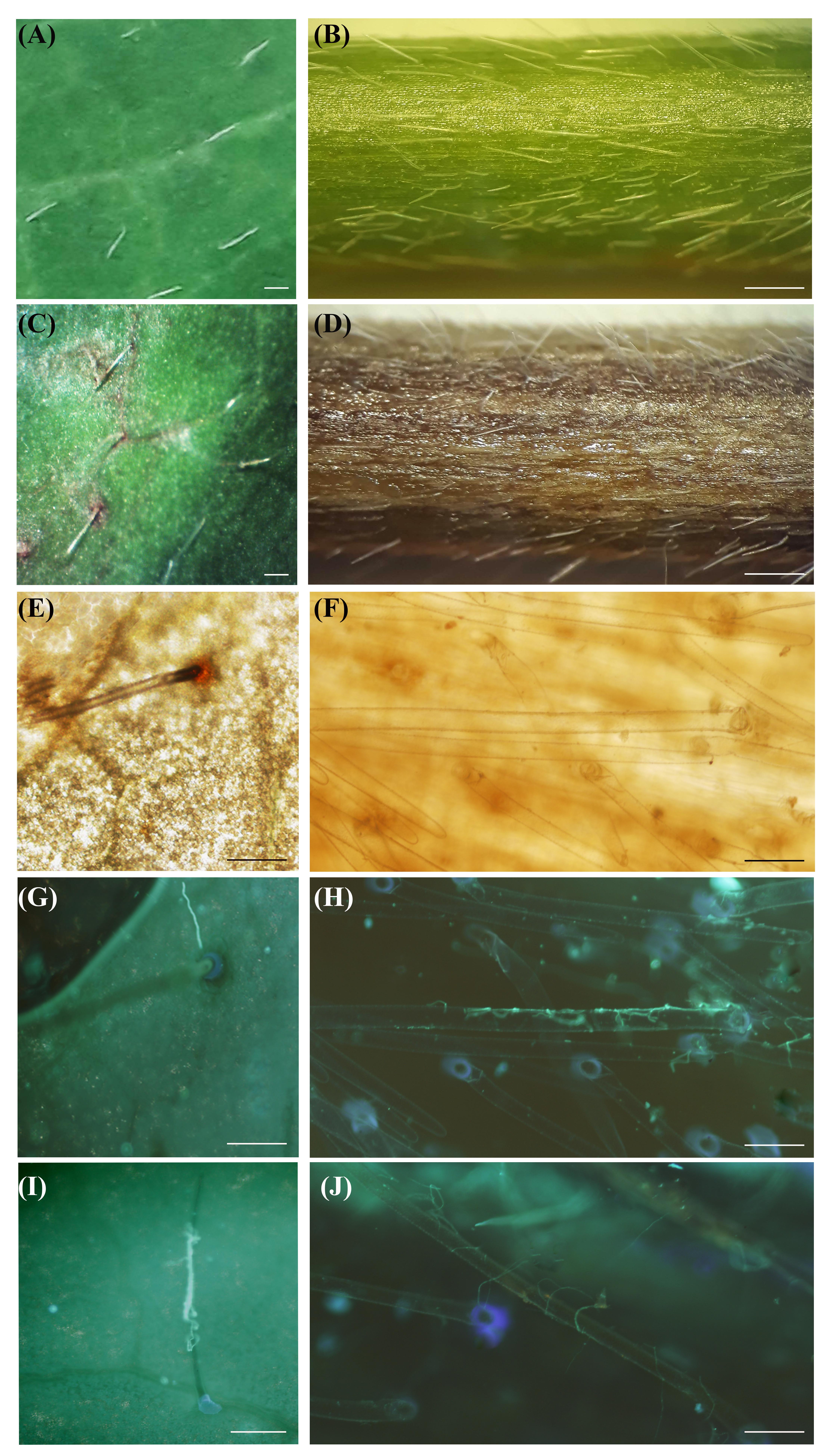

Supplement: Supplementary file 8 [file Image_8.jpeg]

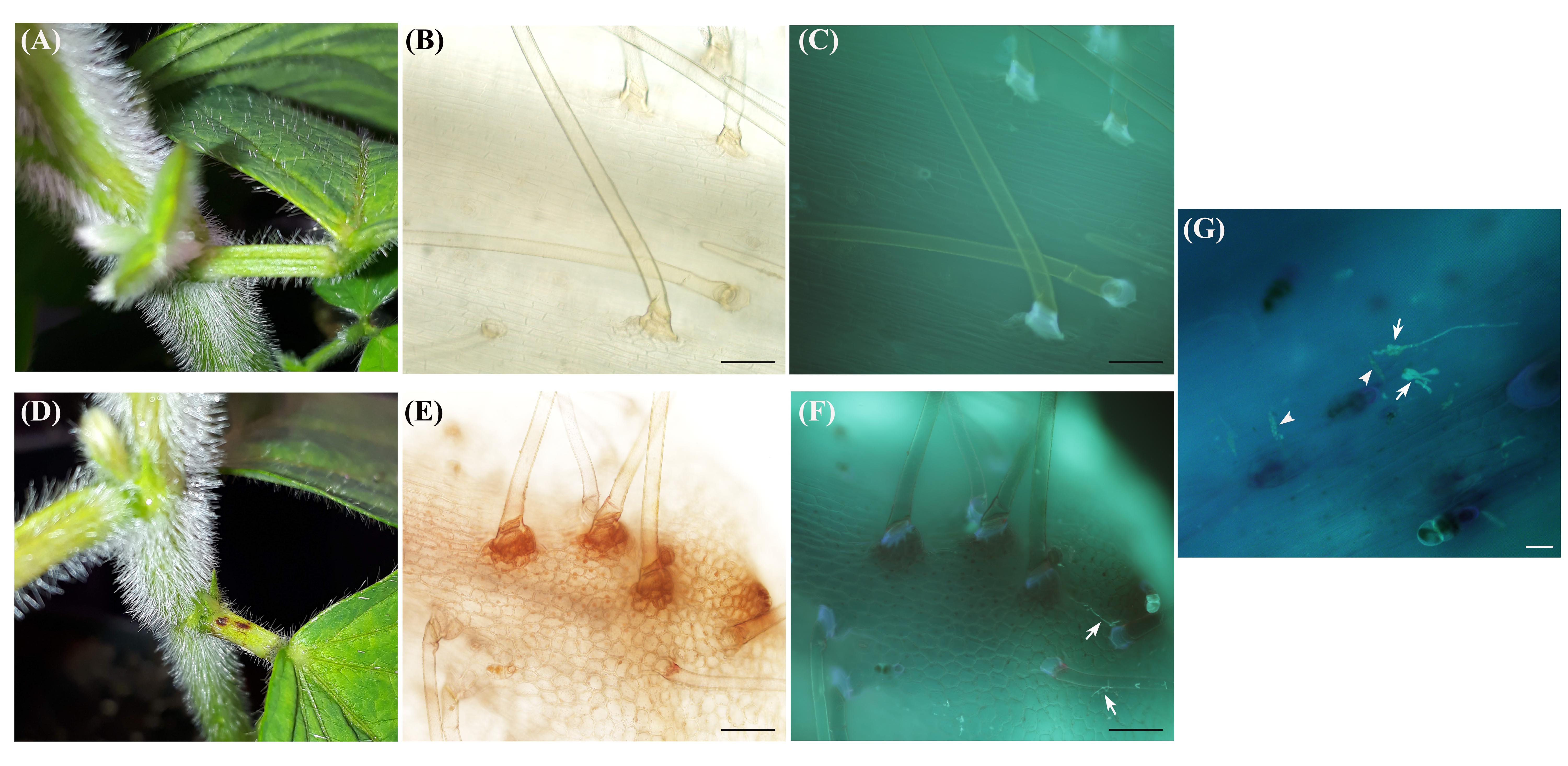

Supplement: Supplementary file 9 [file Image_9.jpg]

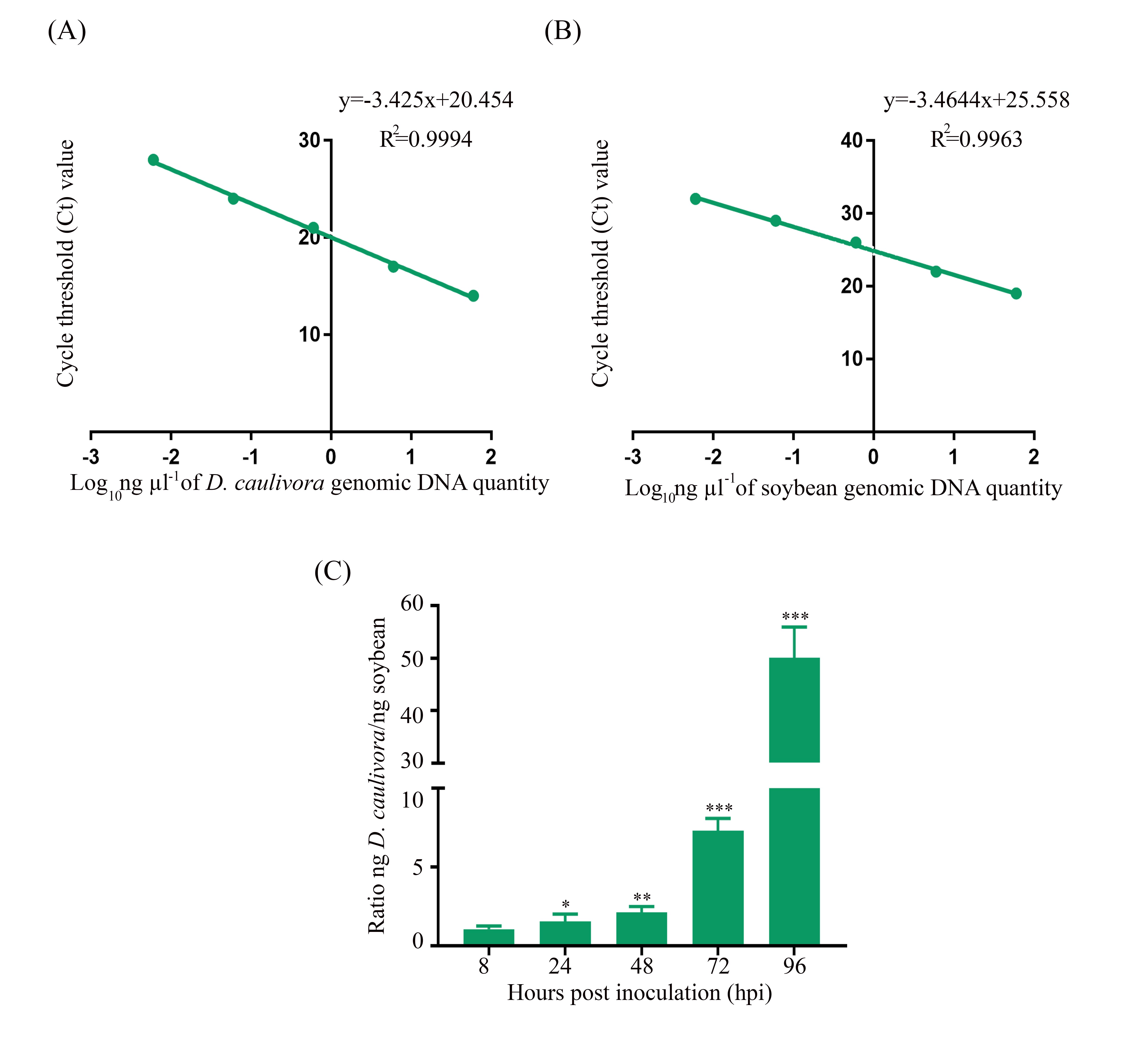

Supplement: Supplementary file 10 [file Image_10.jpg]
